# The role of strategic visibility in shaping wayfinding behavior in multilevel buildings

**DOI:** 10.1038/s41598-024-53420-6

**Published:** 2024-02-14

**Authors:** Michal Gath-Morad, Jascha Grübel, Koen Steemers, Kerstin Sailer, Lola Ben-Alon, Christoph Hölscher, Leonel Aguilar

**Affiliations:** 1 Cambridge Cognitive Architecture, Department of Architecture, University of Cambridge, Cambridge, United Kingdom; 2 Chair of Cognitive Science, ETH Zürich, Zürich, Switzerland; 3 The Behavior and Building Performance Group, Department of Architecture, University of Cambridge, Cambridge, UK; 4 The Space Syntax Laboratory, The Bartlett School of Architecture, University College London, London, UK; 5 Geo-information Science and Remote Sensing Laboratory, Wageningen University, Wageningen, The Netherlands; 6 Game Technology Center, ETH Zürich, Zürich, Switzerland; 7 Visual Computing Group, Harvard University, Cambridge, USA; 8 Center for Sustainable Future Mobility, ETH Zürich, Zürich, Switzerland; 9 Geoinformation Engineering Group, ETH Zürich, Zürich, Switzerland; 10 Graduate School of Architecture, Planning and Preservation (GSAPP), Columbia University, New York, USA; 11 Data Science, Systems and Services Laboratory, ETH Zürich, Zürich, Switzerland

**Keywords:** Human behaviour, Psychology and behaviour

## Abstract

In this paper, we explore the mutual effect of prior background expectations and visibility afforded by the 3D configuration of the physical environment on wayfinding *efficiency* and *strategy* in multilevel buildings. We perform new analyses on data from 149 participants who performed six unaided and directed wayfinding tasks in virtual buildings with varying degrees of visibility. Our findings reveal that the interaction between visibility and prior background expectations significantly affects wayfinding efficiency and strategy during between-floor wayfinding tasks. We termed this interaction effect *strategic visibility*, which emphasizes the importance of the strategic allocation of visibility towards actionable building elements in promoting efficient wayfinding and shaping wayfinding strategy. Our study highlights the significance of *strategic visibility* in promoting inclusive and accessible built environments for neurodiversity. Finally, we provide an open-source dataset that can be used to develop and test new wayfinding theories and models to advance research in the emerging field of human-building interaction.

## Introduction

Human-building interaction design during the architectural design process plays a critical role in supporting efficient and effective *wayfinding*^1,2^—the complex cognitive operation that involves monitoring external and internal cues, forming internal representations of space, and updating them to find one’s way in an environment. The study of wayfinding has been a subject of research for over 60 years, with contributions from a variety of fields, including architecture and urban design^3–7^, psychology^8–14^, neuroscience^15–18^, transportation^19,20^, and computer science^21–24^. Recent trends, such as intense urbanization leading to more complex buildings and cities, the rise of information and communication technologies, an aging population, and a growing awareness of inclusive design, have further fueled research in this area^3,25–31^. It’s important to note that inclusive design also involves considering the needs of individuals with diverse cognitive abilities, such as those with dementia, autism, dyslexia, or ADHD. This concept is referred to as neurodiversity^32,33^, which recognizes neurological differences as a natural part of human diversity. Wayloosing and disorientation can have particularly negative impacts on individuals with cognitive differences, highlighting the importance of inclusive design in creating environments that support all individuals^34^.

To understand the factors that affect wayfinding performance, researchers have classified wayfinding tasks according to the source of information used, the existence or absence of a final destination, the complexity of the search space, and the familiarity with the navigation environment^35^. In this paper, we focus on *unaided* *wayfinding *in *unfamiliar multilevel* environments, which is a common scenario in which people find their way between floors in large search spaces^36,37^. Given the growing complexity of multilevel public buildings in cities, people are often required to wayfind between-floors in large ‘search spaces’ that span horizontally and especially vertically^36,37^.

Efficiency and search strategy are the two main dimensions for measuring wayfinding performance in these contexts^38^. Efficiency can be assessed by measuring task completion rate, duration, distance covered, or average speed. On the other hand, wayfinding strategies are often captured using ‘think aloud’ protocols^10^. Former studies have identified exhaustive search strategies like the *perimeter strategy* (moving along the boundary of an area to reduce the chance of revisiting the same space)^39^, the *lawnmower strategy* (proceeding in straight parallel lanes^40^), and the *directed random search* strategy (selecting turns with the lowest likelihood of retracing one’s former location)^35^.

In more specific contexts, such as retail environments like supermarkets, shoppers tend to exhibit less exhaustive search strategies. Most shoppers prefer to travel along selected aisles and may favor a perimeter strategy, using the store’s perimeter as the main thoroughfare, with occasional trips into the aisles^41^. However, it’s essential to highlight that individual differences among shoppers play a significant role in influencing the choice of search strategy in retail settings. A study of shoppers behavior in a supermarket setting^42^ where products are evenly distributed in a regular grid revealed the existence of distinct clusters of shopping strategies correlated with specific shopper profiles, suggesting that individuals with different backgrounds and preferences tend to adopt different ways of navigating and searching for products within the supermarket. In complex, multi-level buildings with limited visual cues and numerous movement options, additional wayfinding strategies are observed, including the *central point strategy* (staying in public and visually connected areas even if it involves detours), the *direction strategy* (prioritizing minimizing horizontal distance to the estimated target location, regardless of vertical changes), and the *floor strategy* (prioritizing minimizing vertical distance to the target, irrespective of its horizontal position)^10,43,44^.

Prior research on unaided wayfinding in buildings highlights the independent roles of *visibility* and of *prior background expectations* to facilitate wayfinding efficiency and inform the choice of search strategy. The spatial configuration of buildings has a direct impact on visibility, which refers to the degree to which different parts of the environment can be observed from a specific viewpoint^11^. Architectural features such as walls and *atria* can act as occlusions or visibility enablers, respectively. The volumetric configuration of buildings determines what and how much can be seen from different viewpoints^6,10,11,45–47^. Depending on the specific wayfinding task, the role of visibility may be more or less critical. For example, a person navigating a familiar building is likely to rely on their memory rather than visibility^48^. However, depending on the destination and the prior background expectations it evokes, different environmental cues may be perceived as more or less important^49^. In their study, Frankenstein and colleagues^49^ found that different destinations trigger different background expectations regarding the association of environmental cues with the location of the destination. For instance, finding an auditorium, a main exit, or a restroom is associated with more central and public locations, while destinations such as a rear exit, the entrance to the cellar, or a broom closet are associated with more peripheral locations.

Critically, despite findings that emphasise either the role of *visibility* or of *background expectations* during wayfinding, the interaction between them during unaided and directed wayfinding in multilevel buildings is still unclear, both with respect to wayfinding efficiency and wayfinding strategy. Table 1 presents an overview of empirical studies focused on wayfinding in built environments. Each study is analyzed with respect to five main categories; Environment, Experiment, Implementation, Participants and Analysis. As can be seen, the majority of studies have been limited to analyzing *Within-floor wayfinding* tasks (even if the building is multilevel) in which the origin and destination are on the same floor^6,18,38,40,49–62^. In contrast, despite the ubiquity of multilevel public buildings, very few studies have empirically studied the role of visibility or background expectations during *Between-floor wayfinding* tasks in which the origin and the destination are located in different floors^10–12,14,43,44,63–71^. Amongst these studies, only a few experiments observed directed wayfinding (i.e., towards a specific destination) in unfamiliar environments which necessarily involves search^10–12,43,44,64,65,67,68^. This gap limits our understanding of how the architectural design of everyday search environments such as buildings affects wayfinding efficiency and strategy.

**Table 1:**
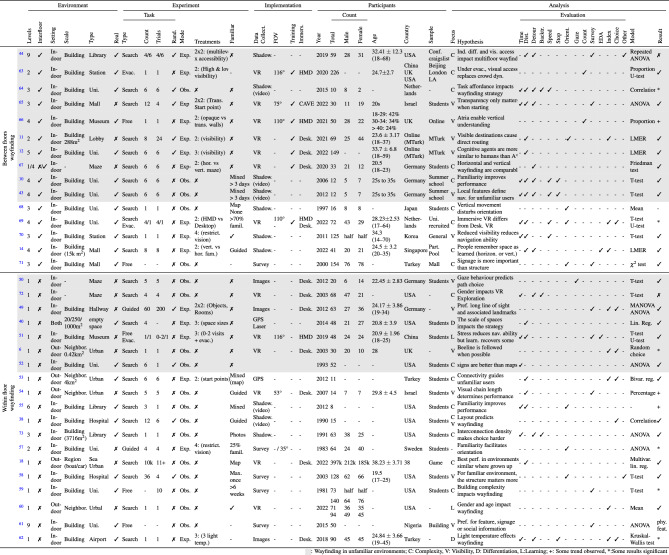
*Literature review.* Comparison of measurements of familiarity and multilevel building in navigation research.

In Grey: Wayfinding in unfamiliar environments; C: Complexity, V: Visibility, D: Differentiation, L:Learning; +: Some trend observed, *:Some results significant.

In this paper, we aim to investigate the mutual role of both *visibility* and *prior background expectations* in unaided wayfinding behavior in unfamiliar multilevel environments. Specifically, we seek to address the question of whether it is primarily the visual cues that affect wayfinding behavior, or whether prior knowledge and expectations also play a role. To this end, we conduct novel analyses on a wayfinding dataset collected in a previously conducted Virtual Reality (VR) wayfinding experiment^12,74^, which has thus far been used as benchmark data to validate a cognitive agent model. In contrast, we focus on the behavioral data collected and investigate the independent and interacting effects of visibility and prior background expectations on wayfinding in unfamiliar multilevel environments.

In the conducted experiment^12^, a total of 149 participants were randomly assigned to one of three building conditions; *Base-case *(control condition), *Atria*, and *Glass* (visibility treatments), see Figs. 8 and 9. The control condition (i.e., *Base-case*) featured a multilevel building with two staircases enclosed in two multi-story cores with concrete walls, resulting in the lowest degree of between-floor visibility. Both the *Atria* and *Glass* treatments were identical to the control building, except for a systematic visibility treatment applied to both buildings, which increased the degree of between-floor visibility compared to the control condition. This visibility treatment involved systematic design interventions applied to the architectural configuration of each building. Specifically, the *Atria* treatment condition involved the creation of six small atria on the second floor, while in the *Glass* treatment condition, the facade of the two circulation cores was replaced from concrete to glass. As part of the experiment, participants in all three conditions were required to wayfind from the same starting position (i.e., the entrance to the building) to six typical building destinations (*i.e., Auditorium, Reading Area, Study Area, Office, Patio, or Roof Terrace*). Although both design variations increased the degree of visibility compared to the control condition, they differed in the type of information being revealed, either the functionality of the second floor or the circulation elements in the building.

Accordingly, we expected that: (H1) wayfinding efficiency and strategy would be affected by the background expectations people have regarding the location of each destination, which may be informed by their previous wayfinding experience; (H2) the *Glass* treatment, which reveals the location of the stairs, would have a significant effect on wayfinding distance performance, resulting in more efficient wayfinding when compared to the other visibility treatment (i.e., *Atria*) or to the control condition (i.e., *Base-case*); (H3) the *Glass* treatment would have a significant effect on the type of wayfinding search strategy employed, resulting in increased *Between-floor* search as opposed to *Within-floor* search when compared to the other visibility treatment (i.e., *Atria*) or to the control condition (i.e., *Base-case*); and (H4) the distribution of paths would be significantly different between the *Glass* condition and the two other building conditions (*Base-case* and *Atria*). To test these hypotheses, we computed several novel wayfinding measures for unfamiliar multilevel buildings based on previously collected raw wayfinding data^12,74^, resulting in the generation of a new wayfinding dataset^75^. All measures capture different aspects of wayfinding efficiency, strategy, and visual perception in an unfamiliar multilevel environment.

Our findings confirm our hypotheses, showing for the first time that there is an interaction effect between *visibility* and *background expectations* during Between-floor search tasks. We name the interaction effect *strategic visibility* and illustrate how it can be applied as an architectural design principle to enhance legibility, thus promoting positive human-building interactions by architecture.

## Results

Our analysis examined the impact of *visibility* and *background expectations* on wayfinding efficiency and wayfinding strategy through two independent variables. The first independent variable we considered was the *Visibility-Treatment* applied to the building (*Base-case*, *Atria*, *Glass*), as shown in Fig. 8. The second independent variable was the wayfinding *Tasks*, which varied based on the destination (*Auditorium, Reading Area, Study Area, Office, Patio, or Roof Terrace*), each assumed to invoke different background expectations.

Our Linear Mixed Effects Regression (LMER) analysis^76^ supported all hypotheses concerning the effect of the *Visibility-Treatment* and *Task* (associated with different background expectations) on both wayfinding strategy and efficiency. Detailed results of this analysis are presented in Table S5 and Table S6, which can be found in the Supplementary Materials S2.

**Figure 1:**
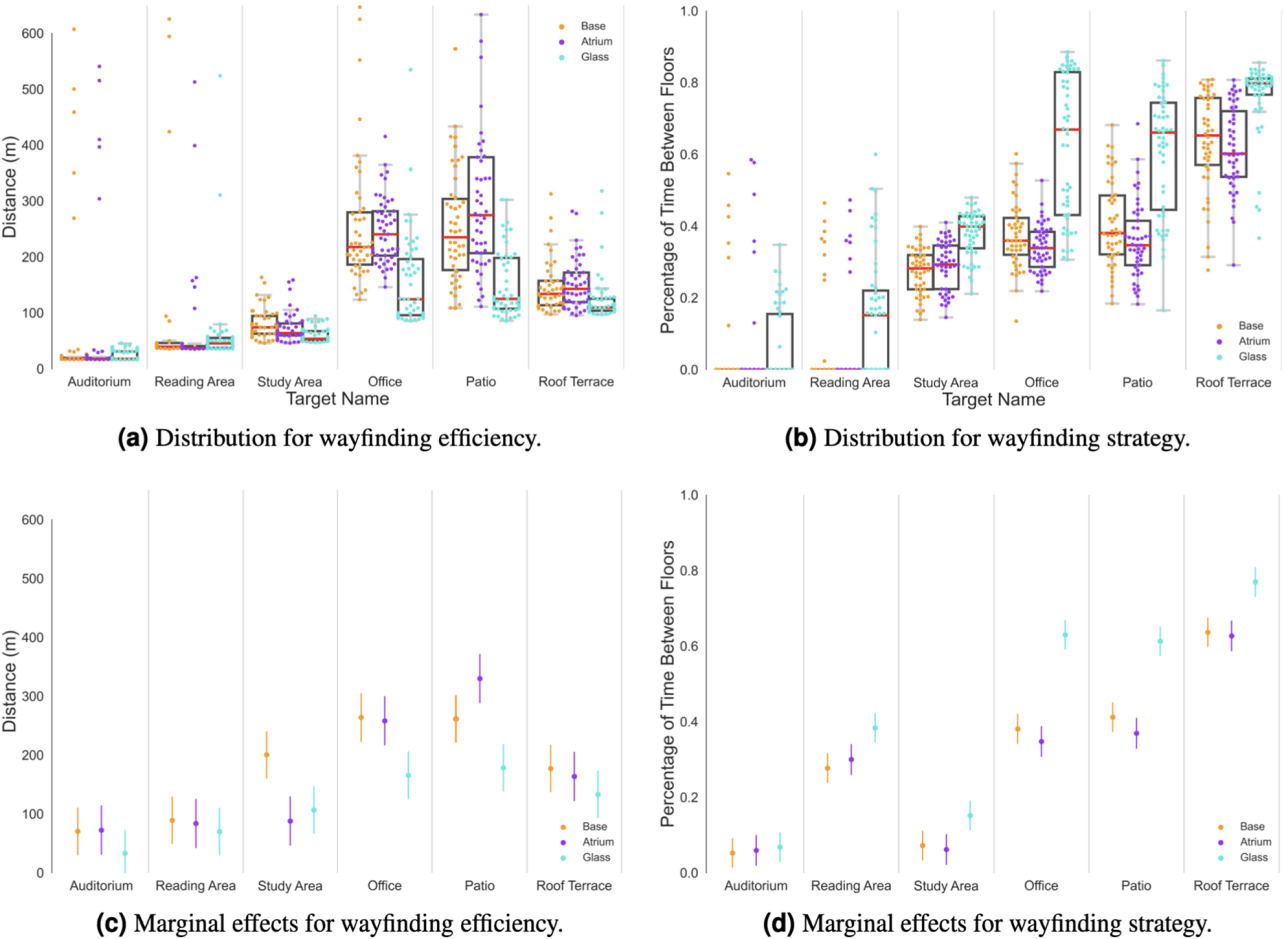
*The effect of Visibility Treatment and Tasks on wayfinding efficiency and strategy.* The *Glass* treatment shows significant differences in the Marginal Effects at the Mean (MEM) for most tasks.

Interpreting models with interaction effects can be challenging since the coefficients cannot be viewed independently. Thus, we calculated the marginal effects of the wayfinding outcome variables to understand the impact of the *Visibility-Treatment* and *Task* (corresponding to different background expectations) on the model results^77^. Specifically, we calculated Marginal Effects at the Mean^78^ for the average response across all trials to gain insights into the effect of *Tasks* and *Visibility-Treatment* on wayfinding efficiency and strategy, See Fig. 1. Additionally, we looked at Average Marginal Effects^79^ for group-level responses. For more detailed information on the analysis methods used, please see the Methods section.

### Wayfinding efficiency

With regards to wayfinding efficiency (see Table 2), the AME shows that the *Glass* treatment has a substantial and significant effect on *Total Distance* performance, resulting in participants walking in average 64.82 meters less across all tasks when compared to the control condition. As expected, the AME of the *Atria* treatment on distance performance was not significant (participants walked on average 14.63 meters less). At the same time, we see that all *Tasks* except the *Reading Area* significantly differ from the baseline task (i.e., where the destination was the *Auditorium* on the first floor which was visible from the entrance). According to our AME analysis, the effect of the *Glass* treatment on distance performance remains significant, even when accounting for the effect of the task type. These findings confirm (H2).

**Table 2:** Average Marginal Effect (AME) for *Tasks* and *Visibility Treatment* for *Total Distance*.

Term	Contrast	Estimate	Std. Error	Statistic	p-value	Conf. low	Conf. high
Building	Atria–Base	− 14.63	11.87	− 1.23	0.22	− 37.88	8.63
Building	Glass–Base	− 64.82	11.71	− 5.53	0.00	− 87.77	− 41.86
Task	Reading Area–Auditorium	22.72	15.13	1.50	0.13	− 6.93	52.37
Task	Study Area–Auditorium	72.58	15.13	4.80	0.00	42.92	102.23
Task	Office–Auditorium	169.68	15.21	11.16	0.00	139.87	199.49
Task	Patio–Auditorium	197.08	15.13	13.03	0.00	167.42	226.73
Task	Roof Terrace–Auditorium	99.41	15.16	6.56	0.00	69.71	129.12

### Wayfinding strategy

With regards to wayfinding strategy (see Table 3), results show that the *Glass* treatment had a significant and positive effect on the percentage of time the participant spent inside the circulation core (p<0.05), which was not demonstrated in the case of the *Atria* treatment. Remarkably, the *Percentage of Between-Floor Movement* in the *Glass* treatment was 13% more than in the control condition. In the case of the *Atria* treatment, the visibility increase did not have a significant effect on wayfinding strategy when compared to the control condition, resulting in a negligible increase of 1% in the *Percentage of Between-Floor Movement*. These findings confirm that participants in the *Glass* treatment were performing significantly more *Between-floor* search when compared to the control condition where a *Within-floor* search was dominant. These findings confirm (H2) and (H3).

**Table 3:** Average Marginal Effect (AME) for *Tasks* and *Visibility-Treatment* for *Percentage Time* spent *Between-Floors.*

Term	Contrast	Estimate	Std. Error	Statistic	p-value	Conf. low	Conf. high
Building	Atria–Base	− 0.01	0.01	− 1.17	0.24	− 0.04	0.01
Building	Glass–Base	0.13	0.01	10.26	0.00	0.10	0.15
Task	Reading Area–Auditorium	0.26	0.01	18.57	0.00	0.23	0.29
Task	Study Area–Auditorium	0.03	0.01	2.43	0.02	0.01	0.06
Task	Office–Auditorium	0.39	0.01	28.21	0.00	0.37	0.42
Task	Patio–Auditorium	0.40	0.01	28.92	0.00	0.38	0.43
Task	Roof Terrace–Auditorium	0.62	0.01	44.14	0.00	0.59	0.64

The discoverability potential of the target in each task is also driving when participants employ *Between-floor* search for a specific task (see Tables 3 and 5), thus meeting our expectations. We contrast each task to the *Auditorium* as the task where no *Between-floor* search is required. We find significant increased *Between-floor* search strategy for the *Reading Area* (26%), the *Office* (39%), the *Patio* (40%) and the *Roof Terrace* (62%). The *Study Area* task does not increase the *Between-floor* search significantly. The discoverability potential of the *Roof Terrace* motivates most participants to opt for a *Between-floor* search strategy whereas the more defuse discoverability of the *Reading Area* only slightly induces this behaviour. The *Office* and *Patio* are somewhat expected to be higher up in terms of discoverability and therefore induce the *Between-floor* search strategy more regularly.

### Background expectations

To test the impact of background expectations on wayfinding efficiency and strategy, we revisit both models, see Fig. 1. Across both efficiency and strategy, a similar pattern emerges with a strong task specific effect which is confirmed in Table 2 and Table 3. As can be seen, regardless of the Visibility-Treatment (*Glass, Atria, Base-case*), the Task type (i.e., destination type) has a significant effect on distance performance, except for the case of the *Auditorium* and *Reading area* tasks (see Table 2 and Table 3. These findings confirm (H1).

**Figure 2:**
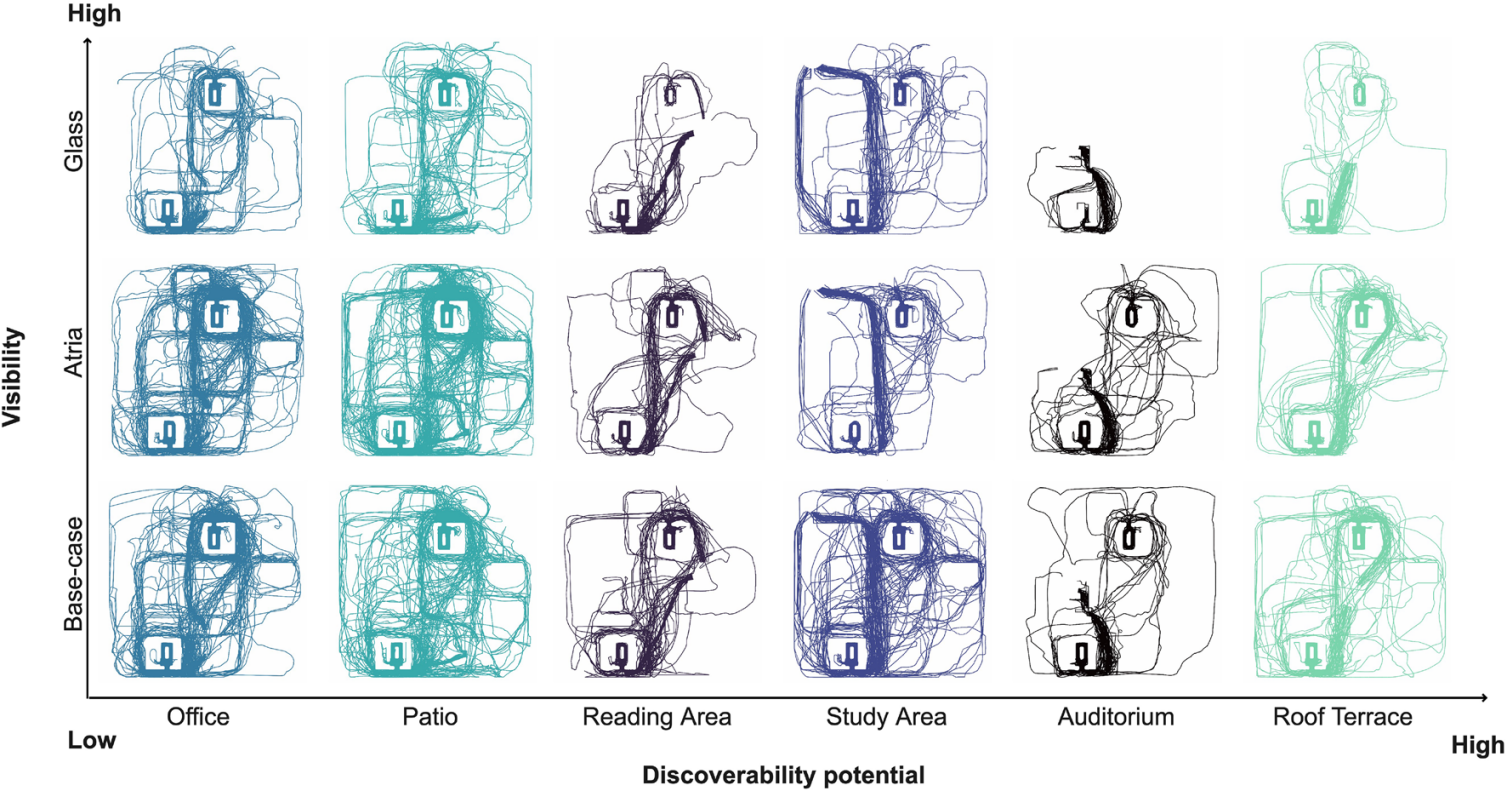
*Trajectories per Task.* Participants’ trajectories for each of the six wayfinding tasks plotted on the same plane across the three building conditions. Comparing trajectories across treatments (rows), participants’ trajectories appear less spread out in the *Glass* treatment (high visibility) compared to the *Base-case* and *Atria* treatments. Comparing trajectories across tasks (columns), the *Auditorium*, *Reading Area*, and *Roof Terrace* visually indicate similar search behavior indicated by the spread of trajectories, possibly related to the prior background expectations participants had regarding the location of these destinations and their *Discoverability Potential*. Similarly, the *Study Area*, *Office*, and *Patio* appear more similar because participants need to explore the floors to find them.

Furthermore, we found that the *Discoverability Potential* of a task (i.e., whether it is low, middle, or high) also impacts wayfinding efficiency and strategy, as demonstrated in Table 5 and Fig. 2. To address concerns that the task structure such as the minimal required walking distance and time could be responsible for these results, we introduced an alternative measure, *Time To Move Up* in the Supplementary Materials describing how much time a participant needed to move up from the first floor. Our analysis shows that it behaved similarly to the other measures.

**Figure 3:**
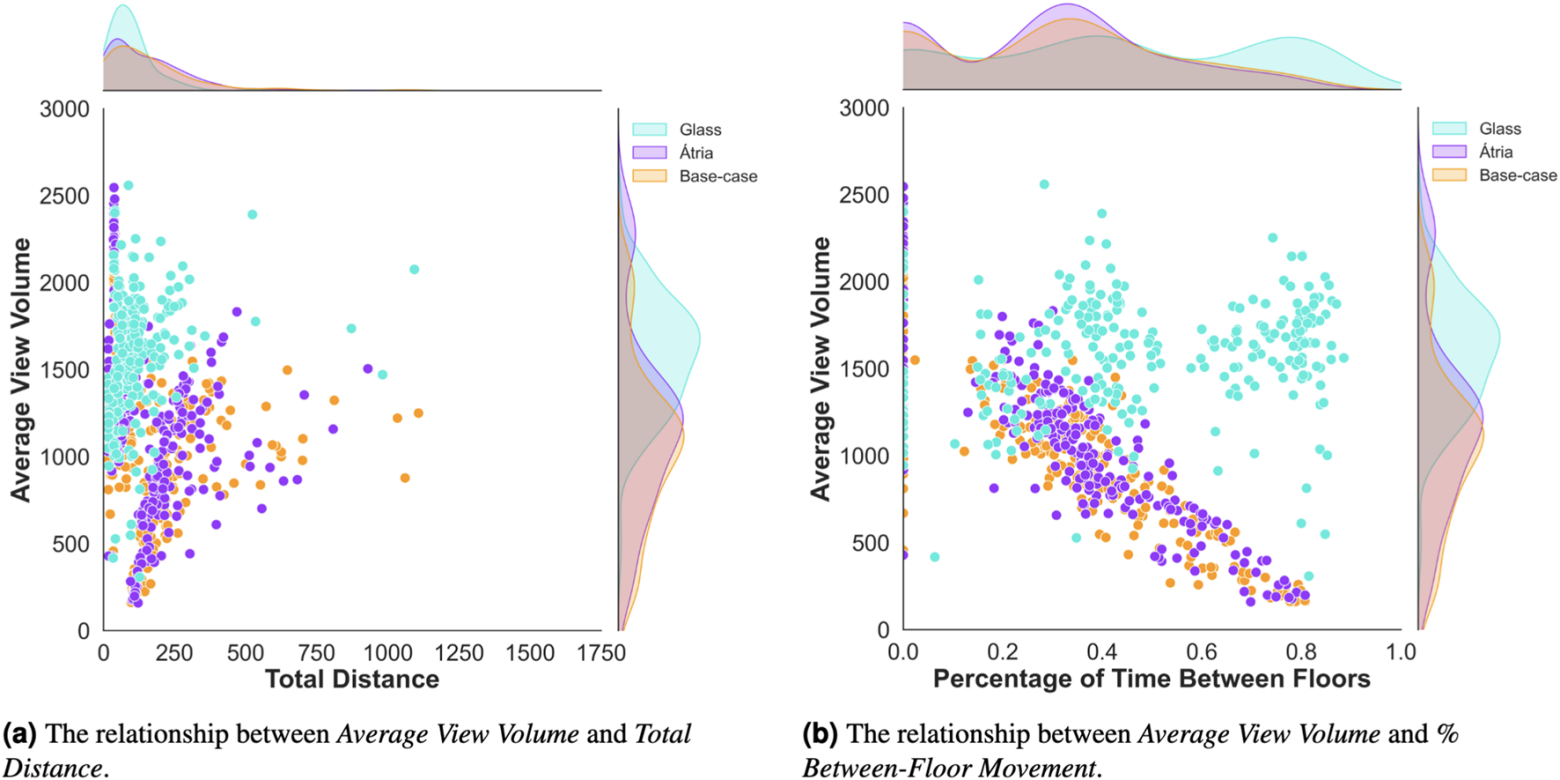
*Average view volume by treatment conditions for wayfinding efficiency and strategy. *Each trial of a participant is represented as a dot combining the amount of visible information (*Average View Volume*) with either wayfinding efficiency (*Total Distance*, or wayfinding strategy *% Between-Floor Movement)*, across building conditions.

### Alternative encoding of visibility treatments

To measure how much visual information participants were gaining in each building condition, we calculated the *Average View Volume* using post-processing on participants’ first-person camera data. The *Average View Volume* was calculated using participants’ first-person camera data to measure the view volume perceived at each path point. By raycasting homogeneous rays from the eye-height of participants towards the environment, the view frustum was calculated, and the view volume at each point was computed. The *Average View Volume* is the mean of these values from all recorded positions between the starting and end point of each trial.

Our analysis presented in Fig. 3 showed that participants in the *Glass* treatment perceived more visual information and had a higher *Average View Volume* compared to the control *Base-case* and *Atria* treatment conditions. However, we found an unexpected relationship between the *Average View Volume* and *% of Between-Floor Movement* in the *Glass* treatment. The visual information gain of participants in this condition was higher, but it remained fairly constant while they were inside the circulation cores.

This finding reinforced the idea that participants’ information gain was not necessarily related to how much more they could see. Instead, persisting with the *Between-floor* strategy in the *Glass* treatment may have been due to the potential for quickly gathering information at a lower cost of angular movement that could have reduced disorientation^6^. We discuss the potential underlying cognitive mechanisms for this phenomenon in the Discussion section. See Fig. 3 for a visual analysis of the relationships between *Average View Volume*, *Total Distance*, and *% of Between-Floor Movement*.

### Differences in spatial distribution of paths

**Figure 4:**
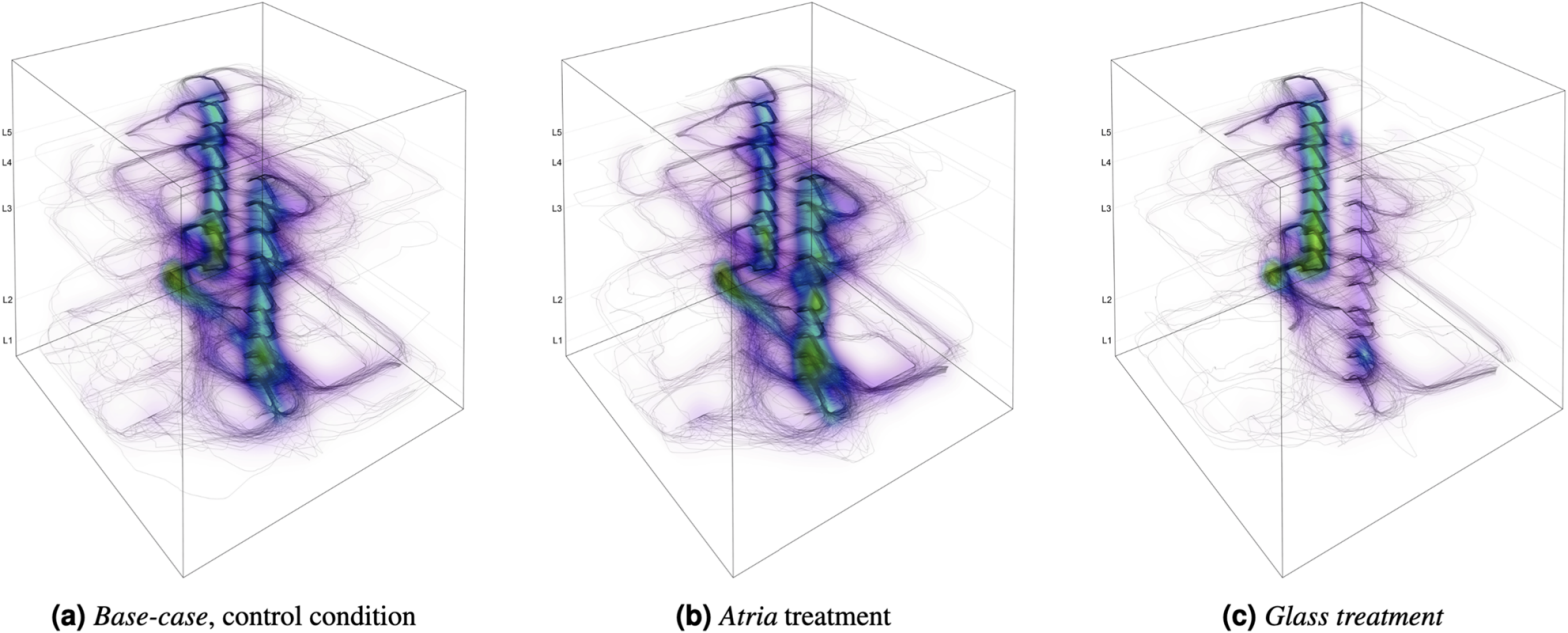
*3D trajectories’ Kernel Density Estimates (KDE) across building conditions*. A comparison of KDE analysis of participants’ trajectories across all tasks and trials between building conditions. The density is scaled from purple (minimal density) over blue (average density) to green (maximal density). The densities are normalized across conditions. The *Base-case* and the *Atria *treatment produce near similar densities. Visually, the *Glass* condition reduces participants’ roaming behavior and focus the participants’ trajectories on using the staircases to find the correct floor.

The visualization in Fig. 2 gives a strong impression of the differences in the spatial distribution of participants’ movement paths across conditions. To further explore these differences, we used Kernel Density Estimation (KDE) to compare the paths in a 3D space, as shown in Fig. 4. A clear pattern emerges as path density in the *Glass* treatment is concentrated around the circulation core that was closer to the starting point, see Fig. 4c. In contrast, we observe more distributed density patterns observed in the *Atria* treatment, see Fig. 4b and the *Base-case* control condition, see Fig. 4a, which appear to be quite similar.

We validate the strong impression provided by the visualization using the KDE test^80,81^. Results confirmed that the *Glass* treatment had a significantly different distribution of trajectories from the other conditions, as seen in Table 4. This provides additional evidence that changing the materiality of the circulation cores from concrete to *Glass* to make the stairs visible nudged participants to move up the stairs. These findings confirm (H4).

Further analysis of *Mean Distance Between Floors* (i.e., indicative of between-floor search) shows that participants in the *Glass* condition significantly favored the front staircase which was closer to the starting position (i.e., origin) of all tasks. In the *Atria* group, while there was a preference for the front staircase, the difference was not statistically significant. In the *Base-case* condition, the back staircase was used slightly more, although not significantly more than the front staircase (see Figure S5 in the Supplementary Materials).

**Table 4:** Comparing KDEs for visibility conditions.

Comparison	T	z	p	$\alpha$*
Base–Atria	156.11	0.20	0.42	0.050
Base–Glass	2409.73	4.71	<**0.025**	0.025
Glass–Atria	2486.71	4.95	<**0.016**	0.016

*Benjamini-Hochberg correction level.

KDE were compared with a non-parametic test^80,81^ (see Supplementary Materials) S3. A significant* p*-value (in bold) implies that the KDE originates from a different distribution. It follows that the *Base-case* condition and *Atria* condition do not differ significantly from each other whereas the *Glass* treatment significantly differs from both.

### Clustering multi-level wayfinding strategies

**Figure 5:**
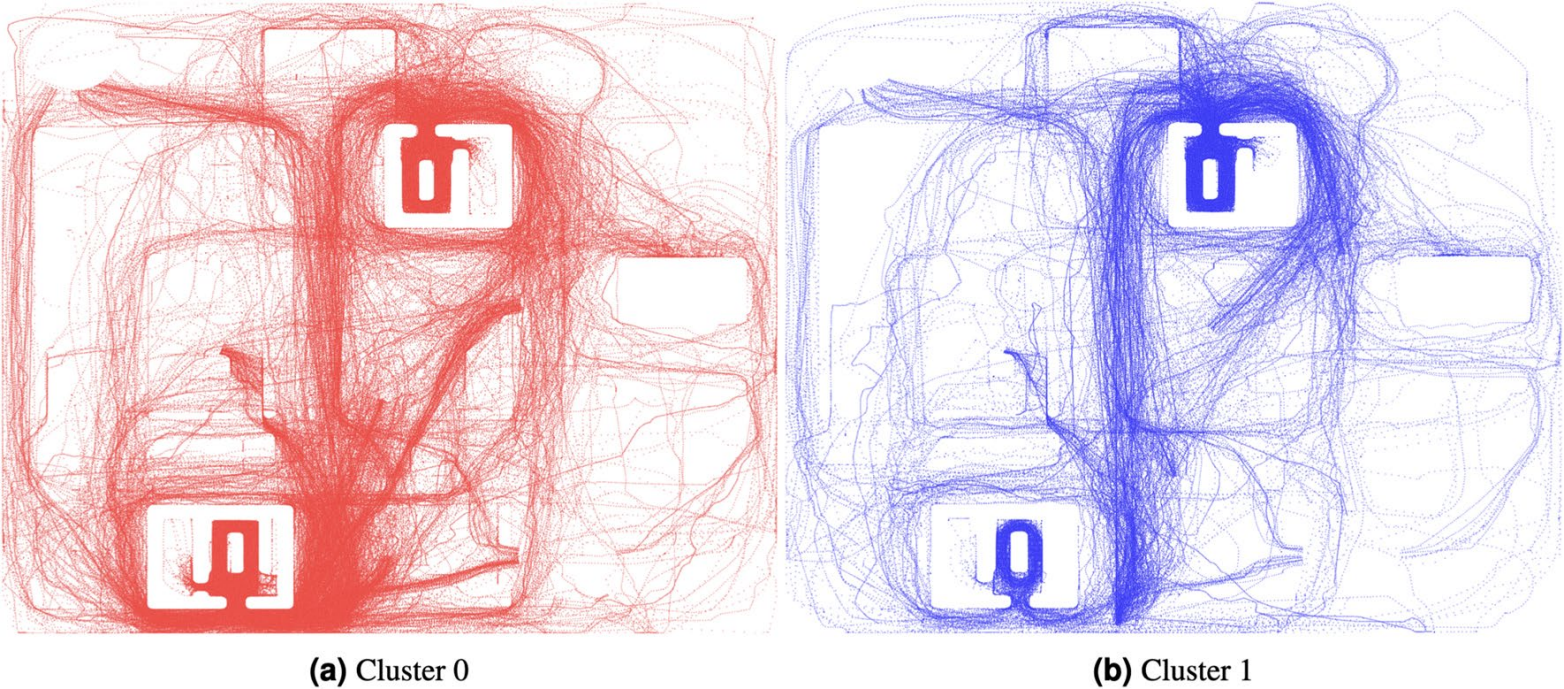
*Spatial distribution of wayfinding paths in two clusters*. A planar projection of wayfinding paths included in either Cluster 1 (red) or Cluster 0 (blue).

**Figure 6:**
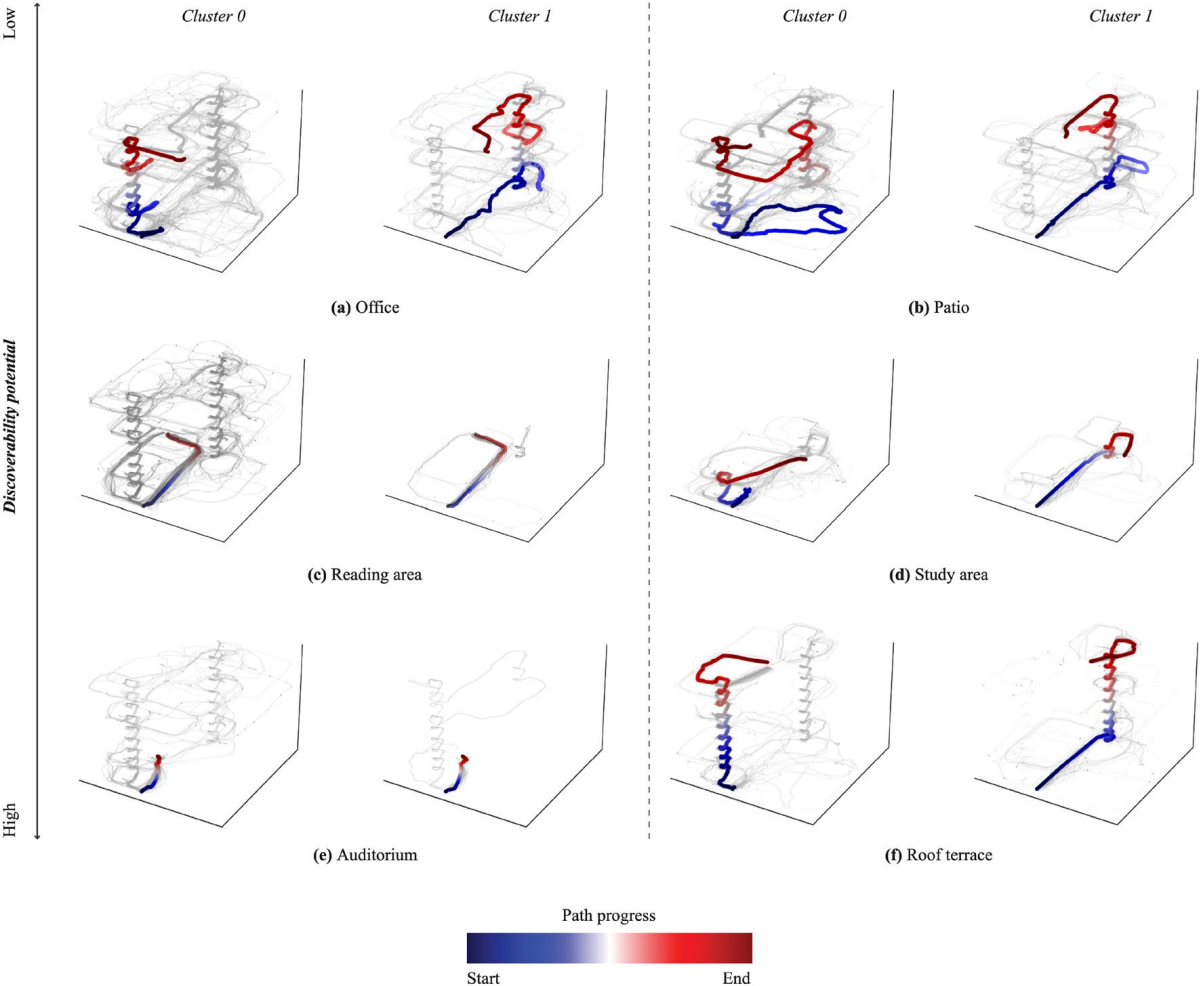
*3D representation of wayfinding paths and progress along path over time*. Paths of representative participants are distinctly highlighted and color-coded to reflect their temporal progress from start (blue) to end (red).

K-means clustering was employed to investigate the impact of individual differences on strategy selection across tasks and building conditions. The input for this clustering process consisted of participants’ spatial wayfinding paths. To ensure comparability between longer and shorter paths, we divided each path into 25 segments, as described in detail in the Methods section. Silhouette scores were used (see Supplementary Materials, Figure S4) to determine the optimal number of clusters for classification. The analysis revealed that clustering into two groups yielded the highest silhouette score of 0.8018 compared to the options with 3, 4, 5, or 6 clusters, indicating meaningful distinctions between participant groups.

**Figure 7:**
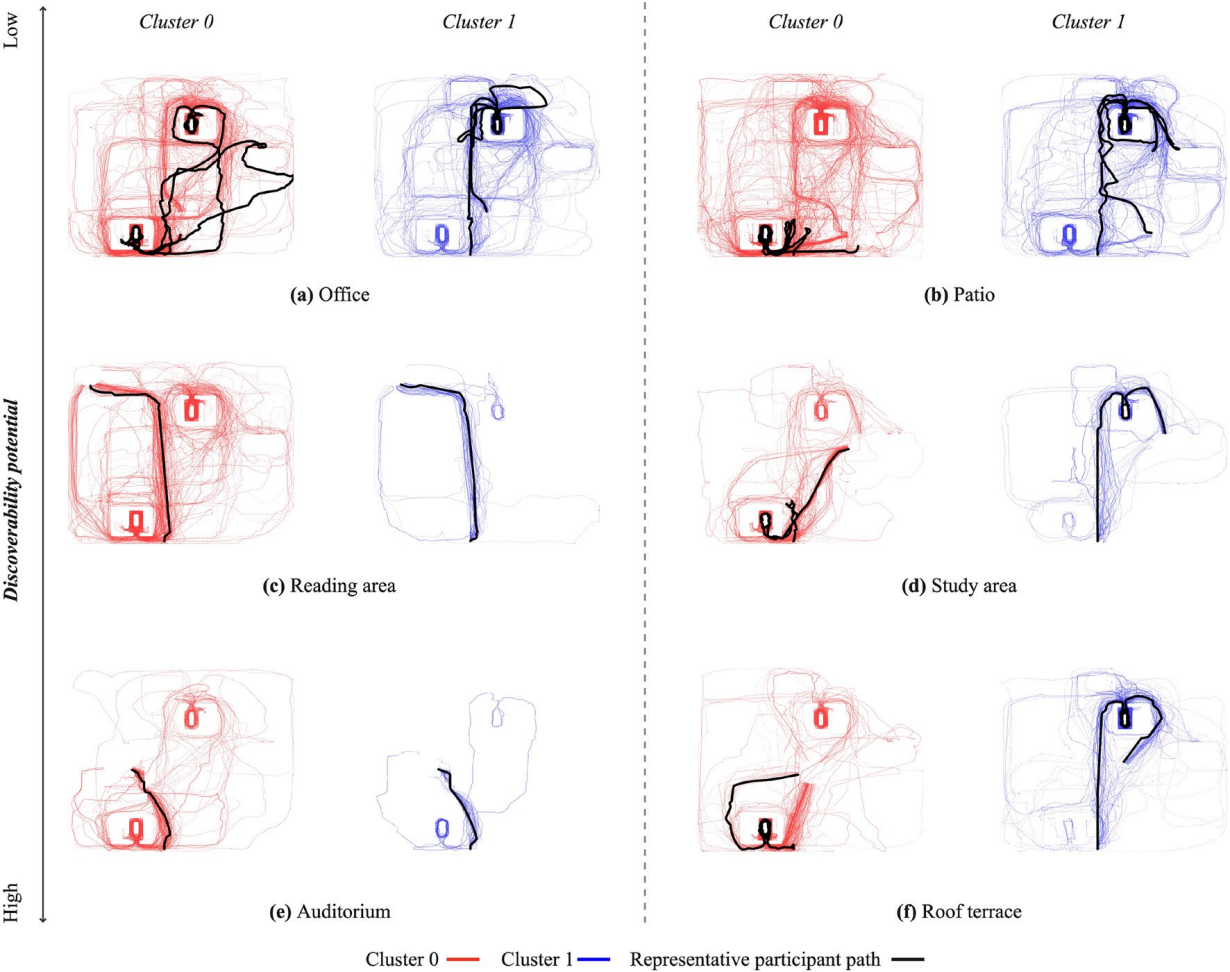
*Two-dimensional projection of wayfinding paths within clusters, with paths of representative participants emphasized in black.*

This analysis resulted in Cluster 0, which included 110 paths, and Cluster 1, which included 36 paths. Figure 5 displays a planar projection of the wayfinding paths in each cluster.

For a more detailed analysis, Figure 7 provides a 2D projection of paths within each cluster for the same task. Additionally, the paths of the most ‘representative participant’ in each cluster, closest to the cluster center coordinates, are highlighted in black. Figure 6 extends the analysis into a 3D representation, with paths colored based on their progress from start to end (blue to red).

A closer examination of paths within each cluster for each task reveals that in Cluster 1, the representative participant consistently favours a *central point search strategy*^10,43^, gravitating towards visually connected areas, even if it involves detours, as evident in all their paths crossing through the centre of the building. For example, in the Roof Terrace task, they initially position themselves in the centre of the floor before ascending the rear staircase. These consistent results across tasks suggest that individual differences are linked to the choice of search strategy. Conversely, in Cluster 0, the representative participant prefers a *perimeter search strategy*^35^, as seen in the *Office, Patio*, and *Roof Terrace* tasks.

It’s noteworthy that for tasks with high or moderate discoverability potential, such as the *Auditorium* or *Reading Area* tasks, where targets are easily visible within a few steps or immediately in the case of the *Auditorium*, the representative participants in both clusters follow identical paths. This suggests that when background expectations and target visibility are high, individual differences in search strategy may be overridden.

## Discussion

The goal of this paper was to investigate the interplay between prior *background expectations* and *visibility* afforded by the volumetric configuration of buildings on wayfinding efficiency and strategy. Our study generated a new dataset^75^ of wayfinding behavior that extends a previous dataset^74^ describing an online desktop VR experiment^12,82^, in which 149 participants performed six unaided and directed wayfinding tasks in one of three unfamiliar multilevel buildings.

These buildings were systematically varied to have either low (*Base-case,* control condition) or high visibility between floors (*Atria*) or towards the circulation elements of the building (*Glass*). Our statistical analysis confirms all four hypotheses. Regarding (H1), our analysis shows that regardless of the visibility treatment applied to the building (*Glass, Atria, Base-case*), the *Task* type (i.e., destination) has a significant effect on distance performance, except for the case of the *Auditorium* and *Reading area* tasks. These findings are in agreement with results from prior studies^49,83^ suggesting that people associate specific environmental cues with the location of unique building destinations.

With respect to (H2) and (H3), our findings demonstrate that greater visibility did not necessarily lead to more efficient wayfinding and did not predict wayfinding strategy, as opposed to prior findings^6^. Our main result shows that the *Glass* treatment, which made the position of the stairs visible from the starting point, led to a significant increase in wayfinding efficiency (participants walked on average 64 meters less than in the *Base*-*case* condition) and predicted the dominant search strategy in the form of *between-floor search*. In addition to confirming hypotheses (H1) through (H3), our statistical analysis also confirms (H4), which predicted that the distribution of paths would be significantly different between the *Glass* treatment and the two other building conditions (*Base-case* and *Atria*). Our analysis shows that the *Glass* treatment led to a significant concentration of path density around the circulation core that was closer to the starting point.

The observed behavior is in good agreement with the principles of Information Foraging Theory (IFT)^84^. IFT borrows concepts from optimal foraging theory in biology^85^ to understand how humans seek information in online environments. The main premise of IFT is that similarly to organisms trying to maximize energy intake while minimizing the cost associated with obtaining it, humans apply similar logic when seeking and interacting with information environments. Borrowing IFT terminology, our findings suggest that in the *Glass* treatment, the ‘information scent’ of the stairs was stronger as a result of increasing visibility, which was not the case in the *Atria* treatment as the ’information scent’ provided was perceived as less relevant to the task. The participants in the *Glass* treatment were able to identify the strategic staircase as the optimal path to their goal due to its increased visibility, and this resulted in a more direct and efficient route to their destination. On the other hand, in the *Atria* treatment, participants may have found it more challenging to locate the staircase, as it was not as strategically visible, resulting in longer and more spread-out routes.

Furthermore, the wayfinding strategies emerging from our analysis align with the high level search strategies described in IFT. If we consider each floor as a potential information patch, human participants performed a *within-patch* search or a *between-patch* search, depending on the strength of the *information scent* emitted by the environment. In the *Glass* treatment, the participants applied a between-patch search strategy, where they quickly identified the location of the most strategically visible staircase and then used it as a landmark to guide their wayfinding behavior. In contrast, the participants in the *Atria* treatment seemed to use a within-patch search strategy, exploring each floor more systematically to locate the less strategically visible staircase.

Results from our clustering analysis shed further light on the potential role of individual differences in the choice of search strategies. In Cluster 1, paths were characterised by more *direct* and *central point-oriented search* strategies^10,43^ indicating a consistent preference for visually connected areas, even if it involved detours. In contrast, Cluster 0 paths favored a *perimeter search strategy*^35^. Intriguingly, for tasks characterised by high or moderate discoverability potential, both clusters converged on similar paths. This suggests that individual differences in search strategy may be overshadowed by task characteristics, highlighting the complex interplay between background expectations, target visibility, and strategy selection.

In addition to these main findings, the observed relationship between *Average View Volume* and participants’ wayfinding behavior was not straightforward. Participants in the *Glass* treatment persisted with the *Between-floor* strategy even though they had a relatively constant visual information gain while inside the circulation cores. This finding suggests that participants’ information gain was not necessarily related to how much more they could see, but rather to a lower cost of angular movement that may result in less disorientation, in accordance with prior wayfinding research in single level environments^6^.

This study makes several important contributions to wayfinding research, evidence-based architectural design and Human-Building Interaction research. Firstly, our findings expand previous research on the impact of visibility on wayfinding^6,10–12,14,18,38,40,43,44,50–71^ and account for the mutual impact of background expectations and visibility on wayfinding. While previous studies have largely focused on the effects of increased visibility on wayfinding, our study highlights the importance of considering peoples’ prior expectations and knowledge of the environment to inform the design of legible building configurations. Secondly, our study highlights the importance of *strategic visibility* in wayfinding. We define *strategic visibility* as the potential of spatial configuration to increase visibility to strategic or actionable elements such as the main staircase of a building. Our results show that *strategic visibility* is a powerful design tool for promoting efficient wayfinding and influencing wayfinding strategy. By using *strategic visibility* to make key wayfinding elements more visible, designers can encourage people to make more informed and efficient wayfinding choices. This has important implications for the design of buildings that promote active living and healthy behaviors, as well as the design of buildings that accommodate people with different abilities and sensory profiles. Thirdly, our study provides an open-source dataset that can be used by other researchers in the broad field of HBI to advance our understanding of wayfinding behavior in complex environments. This dataset contains rich information on peoples’ wayfinding behavior in three different building environments, including their search strategies and distance performance. This dataset can be used to develop and test new wayfinding theories and models, as well as to design and evaluate new wayfinding design strategies.

To the best of our knowledge, this is the first study to analyze the interaction between *visibility* and *prior background expectations* in the context of unaided and directed wayfinding during *Between-floor wayfinding* tasks in an unfamilliar multilevel environment. However, several limitations should be considered when interpreting our findings. Firstly, the dataset used for analysis^75^ was based on wayfinding behavior observed in a Desktop VR setting^12,74^. While evidence suggests that wayfinding in VR and real environments is comparable for strategic decision making^86,87^, the limitations of the behavioural dataset collected in VR should be considered when interpreting our results. Specifically, distance estimation in VR may be less accurate than in reality, and the use of visual motion (as opposed to physical motion) may affect neural encoding of spatial information in memory^88^. Nevertheless, given the design variations included in our treatments, it would have been impossible to conduct a validation experiment in corresponding real-world conditions. Secondly, the random assignment to treatment conditions resulted in an imbalanced sample for gender, with 286 trials in the control group, 294 trials in the Atria treatment, and 310 trials in the *Glass* treatment (see Table S2). While we do not report specific results by gender, an initial review has shown no significant difference^82^,(see Supplementary Materials).

To conclude, our study results demonstrate the crucial interplay between *visibility* and *background expectations* in determining *wayfinding efficiency* and *strategy*. Our findings indicate that the strategic allocation of visibility, rather than mere increased visibility, can be a powerful means of facilitating efficient wayfinding and shaping wayfinding strategy. The alignment of our results with Information Foraging Theory highlights the significance of an environment’s information scent in guiding people’s wayfinding behavior. Practically, our study’s results have implications for HBI research, fueling efforts that promote that design of inclusive and accessible environments catering to individuals with different abilities and sensory profiles, including those on the neurodiverse spectrum. By leveraging the potential of *strategic visibility*, architects can design environments that are more legible, easier to orient in, and more accommodating for all individuals. We hope that our open-source dataset^75^ can serve as a valuable resource for other researchers seeking to deepen our understanding of wayfinding behavior in complex environments, ultimately informing an evidence-based and human-centered approach to design legible built environments in the face of complexity.

## Methods

### The Zollvereine wayfinding study and dataset

#### Study description

The data collection for this wayfinding dataset was conducted as part of a previous VR study^12^ based on an online infrastructure following the *Experiments as Code* paradigm^82,89–91^. For full details, we refer to the previous work above. A concise summary of experimental details relevant to this current study is provided below. In this paper, the Methods description focuses on the hypotheses set and analysis methods used to test it.

**Figure 8:**
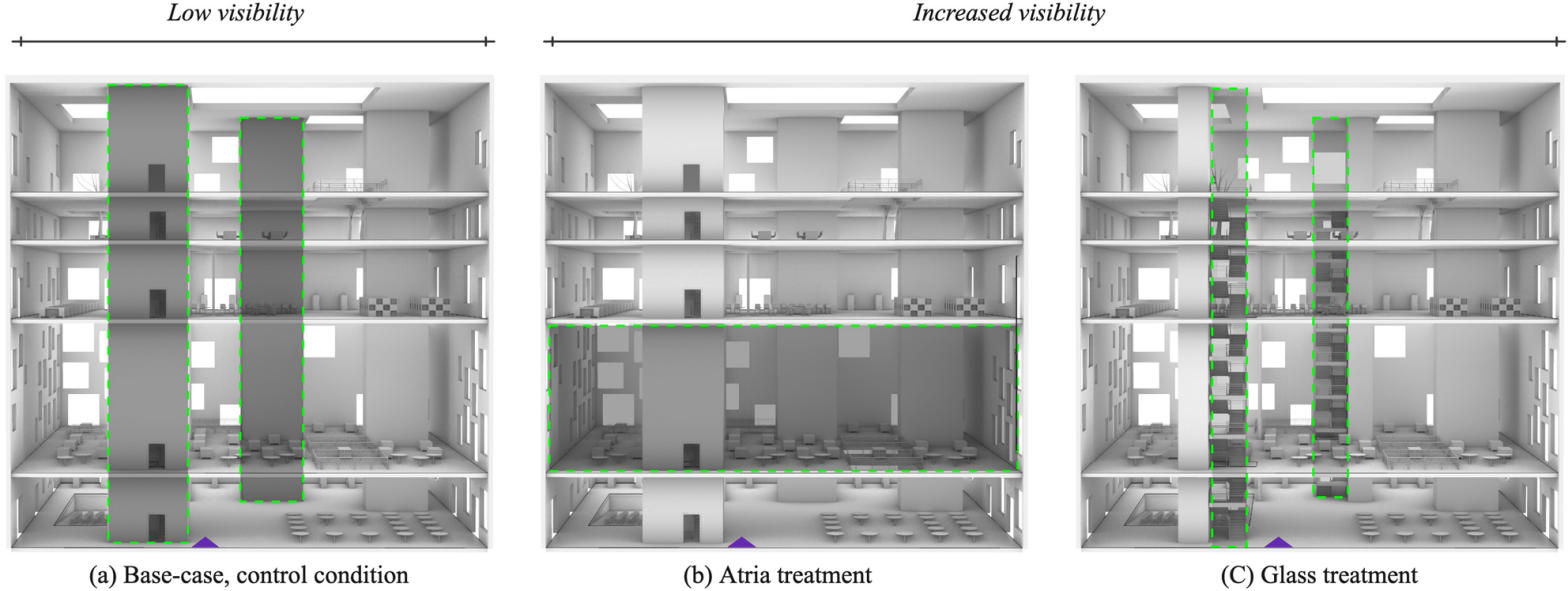
*Perspective elevations for the three building conditions with architectural variations highlighted in a dashed green line.* *Base-case*, the control condition has the lowest visibility with the staircases hidden behind concrete walls and no atria on the second floor. The *Atria* and *Glass* treatments increase visibility either between the first and second floor by means of 6 atria (*Atria)* or towards strategic elements such as the staircases by removing the concrete enclosure on both circulation cores to become transparent (*Glass*). **Legend**: Purple triangle: Origin for all tasks.

**Figure 9:**
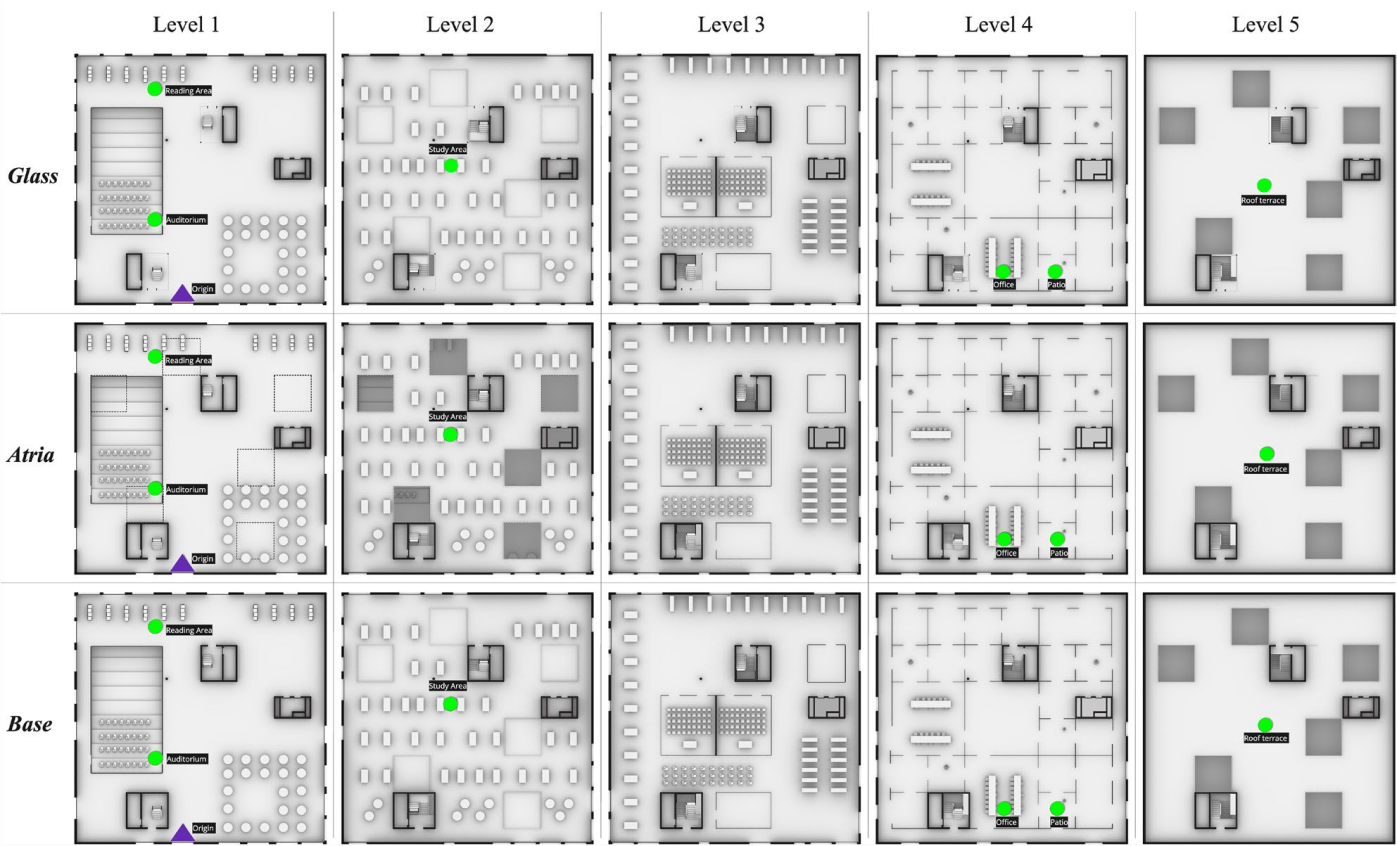
*Floorplans for each level across the three building conditions*; *Base-case*, *Atria*, and *Glass*. Dashed lines on *Atria* Level 1 indicate the locations of six atria on the second floor. On the second floor in the *Atria* condition, views towards the first floor are visible through the six atria. Across levels in the *Glass* condition, the staircase is visible through a glass facade, in contrast to the respective levels in *Base-case* and *Atria*. **Legend**: Purple triangle: Origin for all tasks; Green circle: Destination for each task.

For the original study^12^, 149 participants (44 female, 105 male) were recruited using Amazon’s Mechanical Turk (Mean age = 33.7 years; SD = 6.8 years; Age range = 18 to 59 years). That study was approved by the Research Ethics Committee of ETH Zurich (2020-N-24). Participants signed informed consent before the study and all methods and experiments described in this paper were performed in accordance with the relevant guidelines and regulations. A virtual model of the Zollverein building in Essen (Germany) was generated for the purpose of the VR experiment, referred to as the *Base-case* condition. Two treatments were applied to the *Base-case* condition. These two treatment conditions increase visibility relative to the *Base-case* condition through different architectural design strategies that modify either the building configuration or the building materiality. The three multilevel virtual building models including the control condition and the two treatment conditions are illustrated (see Fig. 9).

**Figure 10:**
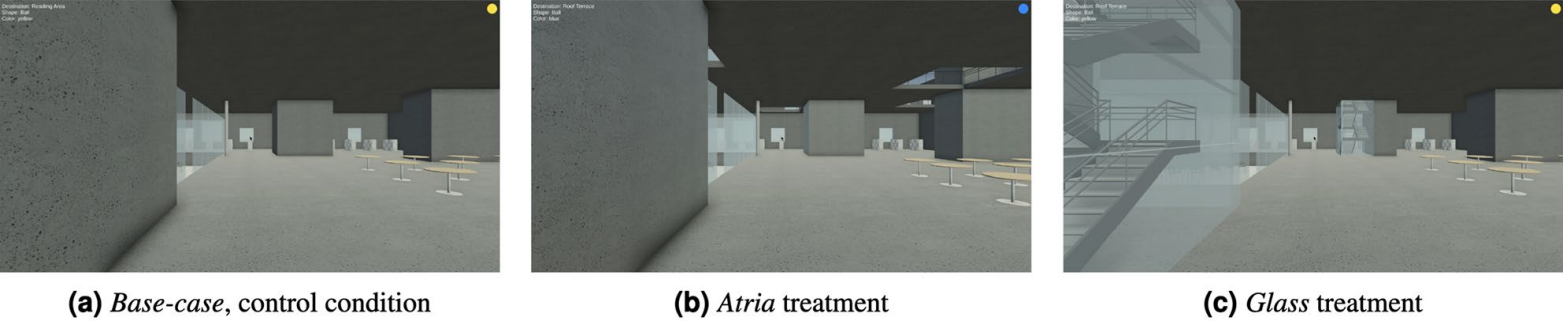
*Participant view in experiment*. An exemplary screenshot from the VR study showcasing the initial first-person perspective (from the same starting position) across the three buildings conditions. In the *Base-case* (left; (**a**)), there is no information available what is behind walls. In the *Atria* treatment (middle; (**b**)), holes in the ceiling partially reveal the floor above. In the *Glass* treatment (right; (c)), the stairs are visible through the glass walls.

For the experiment, participants were randomly assigned to one of the three building conditions. Participants had a first person view of the navigation environment (see Fig. 10). Participants were asked to perform a set of 6 wayfinding tasks (i.e., 1 task per trial). The order of tasks was randomized to avoid practice effects. In each task, they were instructed to find a semantically defined destination (i.e., *Roof Terrace, Patio, Office, Auditorium, Reading Area, Study Area*). Participants were told that the destination would be marked with a colored ball (See Figure S1b) for the instructions given before each wayfinding task). The colour of the ball was randomly generated for each trial. The starting position was located on the ground floor and was the same for all six wayfinding tasks. The six destinations participants had to find were either located within the same floor as the starting position (i.e., *Auditorium* and *Reading Area*), or in one of the upper floors (Level 2: *Study Area*, Level 4: *Office*, *Patio*, and Level 5: *Roof Terrace*). We refer to tasks in which the starting position and destination are on the same floor as ‘within-floor tasks’, and to tasks in which the destination is at a different floor from that of starting position as ‘between-floor tasks’. Tasks were designed to trigger different background expectations, see Table  5. In general, the tasks vary from wayfinding towards highly unique destinations such as an auditorium to more generic ones such as an office. We ranked these destinations according to their *discoverability potential*, see Table 5, following findings from^49^ suggesting that specific building destinations invoke stronger or weaker background exceptions.

**Table 5:** *Overview of background expectations.* Tasks are distributed across different floors and are assumed to raise certain expectations for participants. We also differentiate the tasks expectations according to how locatable they are. *Assumed.

Task	True location	*Expected floor	*Expected horizontal position	*Discoverability potential
Office	Third floor	Undefined	Enclosed, remote	Low
Patio	Fourth floor	Undefined	Central, outdoor	Low
Reading area	Second floor	Close to ground floor	Enclosed, accessible	Medium
Study area	Ground floor	Close to ground floor	Enclosed, accessible	Medium
Auditorium	Ground floor	Ground floor	Central, accessible	High
Roof terrace	Fifth floor	Top floor	Central, outdoor remote	High

#### Dataset description

From the Zollverine experiment, a dataset describing wayfinding behavior in the three building conditions has been generated^74^ and was used in^12^ to validate a cognitive agent model. In this paper, we present an extended and newly annotated wayfinding dataset^75^ used to test several hypotheses concerning the effect of visibility and background expectation on wayfinding behaviour. The new dataset consists of 890 records (rows) and 53 variables (columns). This derivative dataset focuses only on the wayfinding task and includes newly computed variables that are the result of post-processing analysis. In this paper, we focus on analysing a subset of 12 variables presented Table 6. These include metadata on the participants, general task completion information and newly calculated metrics based on visibility and multi-level behaviour. A description of each column in the complete dataset and the overall format of the data are presented in Table S1 and Table S4 in the supplementary materials. Descriptive statistics for the dataset are provided in the supplementary materials (see section S1.2).

**Table 6:** *Variables overview.* This table presents the 12 selected variables for this study extracted from the complete dataset with 890 records and 53 variables that was generated on the basis of the VR experiment raw data by^12^.

Variable	Description
participant	Anonymized participant identifier
task	A semantically defined destination to be reached (e.g., roof-terrace, patio, office, auditorium,reading area, study area)
task_order	The chronological order in which this task was executed
total_distance	Distance walked by the participant in meters
total_time	Total time taken to complete the task in seconds
building	Building condition in which this task was executed (i.e.. Base, Atria or Glass)
age	Age of the participant
gender	Gender of the participant
average_speed	Average participant movement speed inside the VR environment
view_volume_average	Camera view volume minus obstacles limiting this view volume averaged along the path
time_percentage_between_floors	Percentage of time the participant was in the stairs during a task
distance_percentage_between_floors	Percentage of the distance along the participant path that was recorded on the stairs
time_to_stairs	Time in seconds to reach a staircase

To analyze how how visibility and task-related background expectations affected wayfinding efficiency and strategy, specific variables were calculated from the raw data. With respect to wayfinding efficiency, for each trial (i.e., one wayfinding task) the *Total Distance* from the starting point to the destination was calculated. This measure captures the efficiency of finding one’s way, assuming that shorter distances correspond with more efficient wayfinding. With respect to wayfinding strategy, for each trial, the percentage of time the participant spent inside the two circulation cores was also calculated. This measure captures the relative part of the path spent moving between floors. It is also indicative of a ‘between-floor’ search strategy given that the stairs inside the circulation cores are the only means of vertical movement. For simplification purposes, it is assumed that upon entering the circulation cores, participants performed, or intended to perform between-floor movement disregarding their speed (i.e., walking or idle) once inside the cores.

### Analysis

To analyse how visibility and background expectations affect wayfinding efficiency and wayfinding strategy, the main independent variable of interest was the *Visibility-Treatment* applied to the building (*Base-case, Atria, Glass*, see Fig. 9). The wayfinding *Task* was considered a secondary independent variable of interest (i.e., tasks differ with respect to their destination being the *Auditorium, Reading Area, Study Area, Office, Patio, or Roof Terrace*, each of which is assumed to invoke different background exceptions). Based on the literature and our own expectations, the following hypotheses were set *a priori*:

- (H1) Wayfinding efficiency and wayfinding strategy will be affected by the background expectations people have regarding the location of each destination (i.e., wayfinding tasks), resulting in a significant correlation between the relative pattern measures for different destinations across building conditions.
- (H2) The *Glass* treatment revealing the location of the stairs will have a significant effect on wayfinding distance performance, resulting in more efficient wayfinding when compared to the control condition (i.e., *Base-case*). In contrast, the *Atria* treatment will not have a significant effect on distance performance when compared to the control condition.
- (H3) The *Glass* treatment will have a significant effect on the type of wayfinding search strategy employed, resulting in increased ‘between-floor’ search as opposed to ‘within-floor’ search when compared to the control condition. In contrast, the *Atria* treatment will not have a significant effect on wayfinding strategy when compared to the control condition.
- (H4) The distribution of paths will be significantly different between the *Glass *condition and the two other building conditions (*Base-case* and *Atria*).

#### Linear mixed effects regression

We test H1, H2 and H3 through Linear Mixed Effects Regressions (LMER) because participants repeatedly perform wayfinding in the same environment. The random effect allows us to capture the participants’ individual differences^92^ in the data across the different tasks. The LMER use two dependent wayfinding behavioral measures: (1) *Total Distance* and (2) *Percentage of Time Spent Between-Floors*. With regards to wayfinding efficiency (H2), the dependent variable of interest was *Total Distance*. With regards to wayfinding search strategy (H3), the dependent variable of interest was *Percentage of Time Spent Between Floors*. To validate our results we applied a form of triangulation^93^ by choosing analyses with different assumptions^94^. Specifically, we employed different alternative independent variables and explain the choice and results in the Supplementary Materials.

The models for our LMER analysis is as follows:

1 $$Total\;Distance\sim Visibility\;Treatment*{\text{Task}} + Age + Gender + (1|{\text{Participant}})$$

2 $$\% Time\;Between\;Floors\sim Visibility\;Treatment*{\text{Task}} + Age + Gender + (1|{\text{Participant}})$$

Our models test the effects of age, gender and the interaction between visibility treatments and task, fixed effects, on wayfinding efficiency (1) and strategy (2). This allows as to take account of individual differences between participants^95^. We compare our models with potential alternative explaining models in the Supplementary Materials. There, ANOVAs have identify the presented models as best fits. Ignoring the individual differences of participants^92^ with a linear model formulation does not perform better than LMER (see Tab S7). Furthermore, we compare the LMER models to discern whether covariates on age and gender which typically attributed as sources for individual differences^92^ improve our models (see Table S8). We observe that only for *Velocity* and the *Time to Move up,* the models with covariates significantly improve the model fit. These small impact of covariates on the individual performance may be explained by the motivated nature of this experiment^96^ and the non-representative range of the covariates.

The wide variance in task design requires us to analyse the data for each treatment and task jointly as an interaction effect. To overcome interpretability issues of complex models, we opt to represent results as marginal effects. In principle, marginal effects look at how the outcomes changes for different levels of interacting independent variables (covariates). Without interaction terms, marginal effects equal the regression coefficients. However, the term marginal effect is often ill-defined^77^ and therefore we need to clearly delineate which form has been used. We opt for Average Marginal Effects (AME)^97^ and Marginal Effects at the Mean (MEM)^78^ because both are common^98^. MEM is simpler as predicted values for task and visibility treatment are compared to the average response across all respondents. However, the average response may not exist in real data^79^ and is therefore rather abstract in its implication for the real world.

In contrast, AME tries to improve this by calculating a model prediction for all real inputs^99^ instead of the average in MEM. The advantage is that (treatment) groups may present ideosyncracies that are not captured with a global mean of a variable. The averages for the prediction are then constructed for each group and in our case over task and visibility treatment. We compare AME and MEM for our wayfinding efficiency measures in Figure S2 and our wayfinding strategy measures in Figure S3. First, we notice that the overall pattern between visibility treatments remains visually similar across AME and MEM. However, there is a slight difference in values that is visually notable for time and velocity. Nonetheless, the type of marginal effects does not impact the overall outcome. For the models with a significant improved fit with covariates, we investigate the marginal effects and observe, that only for velocity under AME we find a large substantial impact of covariates.

Finally, we are comparing our triangulation measures^93^ to determine whether our effects are robust to differences in measurement. We replace our dependent variable with different measures that we expect to perform similar. First, for wayfinding efficiency, we find that distance and total time have very similar patterns at different scales. Velocity has a different pattern but also shows that glass is different. Distance produces the substantially largest difference between treatment conditions and is selected for the main analysis. Second, for wayfinding strategy, we find all measures produce a similar pattern (with *Time to Move up* being on an inverted scale). The ratio measure has a lower response strength for one task (*Reading Area*) but stronger responses for other tasks (*Office, Patio, and Roof Terrace*). We find that the percentage measure has the smallest confidence intervals and select it for the main analysis. We believe that across measures and methods we can show robust results for our main claims. The robustness of the models and a detailed model comparison with linear models is shown in the Supplementary Materials S2.

#### Kernel density estimates

The trajectories, shown in Fig. 2, are aggregated by visibility treatment into Kernel Density Estimates (KDE) to differentiate physical presence in different sections of the building.

To test H4, we compare these trajectory distributions using a KDE test^80,81^ with the R package ks^100^. Two KDEs are compared with a discrepancy measure^101^ that compares intra-sample differences to inter-sample differences^80^. We test H4 on the KDE of participant trajectories using a closed-form, non-parametric, asymptotically normal, density-based framework^100^, see Supplementary Materials S3 for detailed formulas.

To account for multiple comparisons, we apply the Benjamini-Hochberg correction^102^. This type of correction reduces false discovery rates by applying an increasing penalty depending on the rank of the p-values from highest to lowest.

#### K-means clustering of wayfinding paths

The input for the K-means clustering process comprised participants’ spatial wayfinding paths. To construct feature vectors representing these paths, we followed the methodology outlined in^42^. Specifically, we divided each path into 25 segments and computed the centroid, which includes coordinates (x, y, z), of each segment. This resulted in a detailed feature vector for each participant, encapsulating their path for each of the six wayfinding tasks. These individual task-based feature vectors, consisting of the coordinates of the 25 segments, were then concatenated to create a comprehensive feature vector representing a person’s paths across all six wayfinding tasks. To enhance the clustering performance and reduce dimensionality, this feature vector was projected into two dimensions using UMAP, a technique known for preserving data structure effectively^103,104^. The determination of the optimal number of clusters for the subsequent K-means clustering was informed by silhouette scores (see Supplementary Materials, Figure S4, which assessed the separation of data points within clusters relative to neighbouring clusters. The silhouette scores exhibited variations with respect to the number of clusters, with the highest score observed for two clusters (0.8018), indicating meaningful distinctions between participant groups.

### Ethical approval

The research with human participants was approved by the Research Ethics Committee of ETH Zürich (2020-N-24). The participants were informed on the study goal and gave informed consent and accepted the publication of appropriately anonymised data.

### Supplementary Information

Supplementary Information.

## Data Availability

The open-source extended wayfinding dataset used for analysis is available on^75^.

## References

[1] Becerik-Gerber B (2022). Ten questions concerning human-building interaction research for improving the quality of life. Build. Environ..

[2] Becerik-Gerber B (2022). The field of human building interaction for convergent research and innovation for intelligent built environments. Sci. Rep..

[3] Lynch K (1960). The Image of the City.

[4] Hillier B, Leaman A, Stansall P, Bedford M (1976). Space syntax. Environ. Plann. B Plann. Des..

[5] Benedikt ML (1979). To take hold of space: Isovists and isovist fields. Environ. Plann. B. Plann. Des..

[6] Dalton RC (2003). The secret is to follow your nose: Route path selection and angularity. Environ. Behav..

[7] Emo, B. Wayfinding in real cities: Experiments at street corners. * Proc. International Conference on Spatial Cognition*, 461–477 (Springer, 2012).

[8] Gibson J (1979). The Theory of Affordances the Ecological Approach to Visual Perception.

[9] Passini R (1984). Spatial representations, a wayfinding perspective. J. Environ. Psychol..

[10] Hölscher C, Meilinger T, Vrachliotis G, Brösamle M, Knauff M (2006). Up the down staircase: Wayfinding strategies in multi-level buildings. J. Environ. Psychol..

[11] Gath-Morad M (2021). Visibility matters during wayfinding in the vertical. Sci. Rep..

[12] Gath-Morad M, Melgar LEA, Conroy-Dalton R, Hölscher C (2022). Beyond the shortest-path: Towards cognitive occupancy modeling in BIM. Autom. Constr..

[13] Kuliga, S. *et al.**Developing a Replication of a Wayfinding Study from a Large-Scale Real Building to a Virtual Reality Simulation.* In *Lecture Notes in Computer Science* 126–142 (Springer International Publishing, 2020). 10.1007/978-3-030-57983-8_11.

[14] Mavros, P., van Eggermond, M. & Hölscher, C. Human navigation in a multilevel travelling salesperson problem (2022).

[15] Arbib MA (2012). Brains, machines and buildings: Towards a neuromorphic architecture. Intell. Build. Int..

[16] Jeffery KJ, Jovalekic A, Verriotis M, Hayman R (2013). Navigating in a three-dimensional world. Behav. Brain Sci..

[17] Spiers HJ, Coutrot A, Hornberger M (2022). How the environment shapes our ability to navigate. Clin. Transl. Med..

[18] Coutrot A (2022). Entropy of city street networks linked to future spatial navigation ability. Nature.

[19] Golledge RG (1999). Wayfinding Behavior: Cognitive Mapping and Other Spatial Processes.

[20] Golledge RG, Timmermans H (1990). Applications of behavioural research on spatial problems I: Cognition. Prog. Hum. Geogr..

[21] Raubal, M. & Worboys, M. A formal model of the process of wayfinding in built environments. * Proc. International conference on spatial information theory*, 381–399 (Springer, 1999).

[22] Sohn, S. S., DeStefani, S. & Kapadia, M. Dynamic cognitive maps for agent landmark navigation in unseen environments. *Proc. of the 11th annual international conference on motion, interaction, and games*, 1–6 (2018).

[23] Dubey RK, Thrash T, Kapadia M, Hoelscher C, Schinazi VR (2019). Information theoretic model to simulate agent-signage interaction for wayfinding. Cogn. Comput..

[24] Banino A (2018). Vector-based navigation using grid-like representations in artificial agents. Nature.

[25] Organization, W. H. *Global age-friendly cities: A guide* (World Health Organization, 2007).

[26] Atzori L, Iera A, Morabito G (2010). The internet of things: A survey. Comput. Netw..

[27] Gath-Morad, M., Schaumann, D., Zinger, E., Plaut, P. O. & Kalay, Y. E. How smart is the smart city? assessing the impact of ict on cities. *Proc. International Workshop on Agent Based Modelling of Urban Systems*, 189–207 (Springer, 2016).

[28] Grübel J (2022). The hitchhiker’s guide to fused twins: A review of access to digital twins in situ in smart cities. Remote Sens..

[29] Colombo, G. *et al.* Spatial performance assessment for cognitive evaluation (space): A novel tablet-based tool to detect cognitive impairment (2022). *Proc. 4th Interdisciplinary Navigation Symposium (iNAV 2022). Virtual Meeting*. June 14–16, 2022; Poster abstract.

[30] Heylighen A, Van der Linden V, Ten Van Steenwinkel I (2017). questions concerning inclusive design of the built environment. Build. Environ..

[31] Zallio M, Clarkson PJ (2021). Inclusion, diversity, equity and accessibility in the built environment: A study of architectural design practice. Build. Environ..

[32] McGee M (2012). Neurodiversity. Contexts.

[33] Holt-Damant, K., Guaralda, M., Taylor Gomez, M. & Nicollet, C. Urban jungles: Making cities healthy places for australians with neurodiversity. *Proc. of the 6th Making Cities Liveable Conference in conjunction with Sustainable Transformation Conference*, 116–132 (AST Management Pty Ltd, 2013).

[34] Carpman JR, Grant MA (2002). Wayfinding: A Broad View.

[35] Wiener JM, Büchner SJ, Hölscher C (2009). Taxonomy of human wayfinding tasks: A knowledge-based approach. Spat. Cogn. Comput..

[36] Al S (2016). Mall City: Hong Kong’s Dreamworlds of Consumption.

[37] Frampton A, Solomon JD, Wong C (2012). Cities Without Ground: A Hong Kong Guidebook.

[38] Peponis J, Zimring C, Choi YK (1990). Finding the building in wayfinding. Environ. Behav..

[39] Ruddle RA, Payne SJ, Jones DM (1997). Navigating buildings in “desk-top“ virtual environments: Experimental investigations using extended navigational experience. J. Exp. Psychol. Appl..

[40] Pingel TJ, Schinazi VR (2014). The relationship between scale and strategy in search-based wayfinding. Cartograph. Perspect..

[41] Larson JS, Bradlow ET, Fader PS (2005). An exploratory look at supermarket shopping paths. Int. J. Res. Mark..

[42] Gil, J., Tobari, E., Lemlij, M., Rose, A. & Penn, A. The differentiating behaviour of shoppers: clustering of individual movement traces in a supermarket. *Proc. 7th International Space Syntax Symposium* (Royal Institute of Technology (KTH), 2009).

[43] Hölscher C, Brösamle M, Vrachliotis G (2012). Challenges in multilevel wayfinding: A case study with the space syntax technique. Environ. Plann. B Plann. Des..

[44] Kuliga SF (2019). Exploring individual differences and building complexity in wayfinding: The case of the seattle central library. Environ. Behav..

[45] Heft H (1996). The ecological approach to navigation: A Gibsonian perspective. The Construction of Cognitive Maps.

[46] Norman DA (1993). Cognition in the head and in the world: An introduction to the special issue on situated action. Cogn. Sci..

[47] Schaur E (1991). Unplanned Settlements/Non-planned Settlements.

[48] Afrooz A, White D, Parolin B (2018). Effects of active and passive exploration of the built environment on memory during wayfinding. Appl. Geogr..

[49] Frankenstein J, Brüssow S, Ruzzoli F, Hölscher C (2012). The language of landmarks: the role of background knowledge in indoor wayfinding. Cogn. Process..

[50] Wiener JM, Hölscher C, Büchner S, Konieczny L (2012). Gaze behaviour during space perception and spatial decision making. Psychol. Res..

[51] Lin J, Cao L, Li N (2019). Assessing the influence of repeated exposures and mental stress on human wayfinding performance in indoor environments using virtual reality technology. Adv. Eng. Inform..

[52] Butler DL, Acquino AL, Hissong AA, Scott PA (1993). Wayfinding by newcomers in a complex building. Hum. Factors.

[53] Kubat, A. S., Özbil, A., Özer, Ö. & Ekinoğlu, H. The effect of built space on wayfinding in urban environments: a study of the historical peninsula in Istanbul. *Proc. Eighth International Space Syntax Symposium*, vol. 8029 (2012).

[54] Omer I, Goldblatt R (2007). The implications of inter-visibility between landmarks on wayfinding performance: An investigation using a virtual urban environment. Comput. Environ. Urban Syst..

[55] Li R, Klippel A (2012). Wayfinding in libraries: Can problems be predicted?. J. Map Geograph. Librar..

[56] O’Neill MJ (1991). Effects of signage and floor plan configuration on wayfinding accuracy. Environ. Behav..

[57] Gärling T, Lindberg E, Mäntylä T (1983). Orientation in buildings: Effects of familiarity, visual access, and orientation aids. J. Appl. Psychol..

[58] Haq S, Zimring C (2003). Just down the road a piece: The development of topological knowledge of building layouts. Environ. Behav..

[59] Weisman J (1981). Evaluating architectural legibility: Way-finding in the built environment. Environ. Behav..

[60] Hegarty M (2022). Understanding differences in wayfinding strategies. Top. Cognit. Sci..

[61] Maina, J. J. & Umar, B. Wayfinding in multi-level buildings: A study of the senate building, ahmadu bello university. In *Proc. 6th West Africa Built Environment Research (WABER) Conference*, vol. 2, 1227–1241 (2015).

[62] Suzer OK, Olgunturk N, Guvenc D (2018). The effects of correlated colour temperature on wayfinding: A study in a virtual airport environment. Displays.

[63] Zhu R, Lin J, Becerik-Gerber B, Li N (2020). Influence of architectural visual access on emergency wayfinding: A cross-cultural study in China, United Kingdom and United States. Fire Saf. J..

[64] Vogels, J. *Wayfinding in complex multilevel buildings A case study of University Utrecht Langeveld building*. Master’s thesis, Utrecht University (2015).

[65] Natapov A, Parush A, Laufer L, Fisher-Gewirtzman D (2022). Architectural features and indoor evacuation wayfinding: The starting point matters. Saf. Sci..

[66] Lazaridou A, Psarra S (2021). How do atria affect navigation in multi-level museum environments?. Archit. Sci. Rev..

[67] Bock O, Fricke M, Koch I (2020). Human wayfinding in the horizontal versus vertical plane. J. Environ. Psychol..

[68] Soeda M, Kushiyama N, Ohno R (1997). Wayfinding in cases with vertical motion. Proc. MERA.

[69] Feng Y, Duives DC, Hoogendoorn SP (2022). Wayfinding behaviour in a multi-level building: A comparative study of HMD VR and desktop VR. Adv. Eng. Inform..

[70] Jeon G-Y, Kim J-Y, Hong W-H, Augenbroe G (2011). Evacuation performance of individuals in different visibility conditions. Build. Environ..

[71] Dogu U, Erkip F (2000). Spatial factors affecting wayfinding and orientation: A case study in a shopping mall. Environ. Behav..

[72] Vila, J., Beccue, B. & Anandikar, S. The gender factor in virtual reality navigation and wayfinding. *Proc. 36th Annual Hawaii International Conference on System Sciences, 2003. Proceedings of the*, 7 (IEEE, 2003).

[73] O’Neill MJ (1991). Evaluation of a conceptual model of architectural legibility. Environ. Behav..

[74] Aguilar, L. & Gath-Morad, M. MichalGath/Wayfinding Behavior in MultiLevel Buildings Dataset (v1.0.0) [Data set]. *Zenodo*10.5281/zenodo.5708071 (2023).

[75] Gath-Morad, M. & Aguilar, L. Strategic Visibility and Human Wayfinding in Multilevel Buildings: Extended Dataset, *Zenodo*, Version v1, 10.5281/zenodo.10646029 (2024).

[76] Bates D, Mächler M, Bolker B, Walker S (2015). Fitting linear mixed-effects models using lme4. J. Stat. Softw..

[77] Heiss, A. Marginalia: A guide to figuring out what the heck marginal effects, marginal slopes, average marginal effects, marginal effects at the mean, and all these other marginal things are (2022). https://www.andrewheiss.com/blog/2022/05/20/marginalia/, Last accessed on 2022-08-31.

[78] Lüdecke D (2018). ggeffects: Tidy data frames of marginal effects from regression models. J. Open Sour. Softw..

[79] Hanmer MJ, Kalkan OK (2013). Behind the curve: Clarifying the best approach to calculating predicted probabilities and marginal effects from limited dependent variable models. Am. J. Polit. Sci..

[80] Duong T, Goud B, Schauer K (2012). Closed-form density-based framework for automatic detection of cellular morphology changes. Proc. Natl. Acad. Sci..

[81] Grübel, J., Wise, S., Thrash, T. & Hölscher, C. A cognitive model for routing in agent-based modelling. *Proc. AIP Conference Proceedings*, vol. 2116, 250005 (AIP Publishing LLC, 2019).

[82] Aguilar, L. *et al.* Experiments as code: A concept for reproducible, auditable, debuggable, reusable, & scalable experiments. arXiv preprint arXiv:2202.12050 (2022).

[83] Frankenstein, J., Büchner, S. J., Tenbrink, T. & Hölscher, C. Influence of geometry and objects on local route choices during wayfinding. In *Spatial Cognition VII*, 41–53 (Springer Berlin Heidelberg, 2010). 10.1007/978-3-642-14749-4_7.

[84] Pirolli P, Card S (1999). Information foraging. Psychol. Rev..

[85] Kamil AC (1983). Optimal foraging theory and the psychology of learning. Am. Zool..

[86] Thrash T (2015). Evaluation of control interfaces for desktop virtual environments. Presence Teleoperators Virtual Environ..

[87] Moussaïd M, Helbing D, Theraulaz G (2011). How simple rules determine pedestrian behavior and crowd disasters. Proc. Natl. Acad. Sci..

[88] Hinterecker T (2018). Body-relative horizontal-vertical anisotropy in human representations of traveled distances. Exp. Brain Res..

[89] Grübel, J. *Computer-Aided Experimentation for Human Behaviour Analysis*. Ph.D. thesis, ETH Zurich (2022).

[90] Grübel J, De T (2023). Handbook of Digital Twins. Chap. Experiments as Digital Twins.

[91] Grübel J (2023). The design, experiment, analyse, and reproduce principle for experimentation in virtual reality. Front. Virtual Real..

[92] Wolbers T, Hegarty M (2010). What determines our navigational abilities?. Trends Cogn. Sci..

[93] Munafò MR, Smith GD (2018). Robust research needs many lines of evidence. Nature.

[94] Lawlor DA, Tilling K, Smith DG (2016). Triangulation in aetiological epidemiology. Int. J. Epidemiol..

[95] Hegarty M, Waller DA (2005). Individual Differences in Spatial Abilities.

[96] Schinazi VR (2023). Motivation moderates gender differences in navigation performance. Sci. Rep..

[97] Arel-Bundock, V. *marginaleffects: Marginal effects, marginal means, predictions, and contrasts* (2022). R package version 0.7.0.

[98] Bartus T (2005). Estimation of marginal effects using margeff. Stand. Genomic Sci..

[99] Leeper, T. J. Interpreting regression results using average marginal effects with r’s margins. *Available at the comprehensive R Archive Network (CRAN)* 1–32 (2017).

[100] Duong T (2007). ks: Kernel density estimation and kernel discriminant analysis for multivariate data in r. J. Stat. Softw..

[101] Anderson NH, Hall P, Titterington DM (1994). Two-sample test statistics for measuring discrepancies between two multivariate probability density functions using kernel-based density estimates. J. Multivar. Anal..

[102] Benjamini Y, Hochberg Y (1995). Controlling the false discovery rate: A practical and powerful approach to multiple testing. J. Roy. Stat. Soc. Ser. B (Methodol.).

[103] Allaoui, M., Kherfi, M. L. & Cheriet, A. Considerably improving clustering algorithms using umap dimensionality reduction technique: A comparative study. *Proc. International conference on image and signal processing*, 317–325 (Springer, 2020).

[104] Hozumi Y, Wang R, Yin C, Wei G-W (2021). Umap-assisted K-means clustering of large-scale SARS-COV-2 mutation datasets. Comput. Biol. Med..

