# Supplementary material for: The role of strategic visibility in shaping wayfinding behavior in multilevel buildings
Source: Sci Rep. 2024 Feb 14;14:3735. doi: 10.1038/s41598-024-53420-6 (PMC10866884; doi:10.1038/s41598-024-53420-6)
Supplement: Supplementary file 1 — Supplementary Information. [file 41598_2024_53420_MOESM1_ESM.pdf]

Supplementary Materials: The Role of Strategic Visibility in Shaping Wayfinding Behavior in Multilevel Buildings

Michal Gath-Morad[1,2,3,4*], Jascha Grübel[2,5,6,7,8,9], Koen Steemers[3], Kerstin Sailer[4], Lola Ben-Alon[10], Christoph Hölscher[2], and Leonel Aguilar[2,11]

[1]Cambridge Cognitive Architecture, Department of Architecture, University of Cambridge
[2]Chair of Cognitive Science, ETH Zürich, Switzerland
[3]The Behavior and Building Performance Group, Department of Architecture, University of Cambridge, UK
[4]The Space Syntax Laboratory, The Bartlett School of Architecture, University College London, UK
[5]Geo-information Science and Remote Sensing Laboratory, Wageningen University, The Netherlands
[6]Game Technology Center, ETH Zürich, Switzerland
[7]Visual Computing Group, Harvard University
[8]Center for Sustainable Future Mobility, ETH Zürich, Switzerland
[9]Geoinformation Engineering Group, ETH Zürich, Switzerland
[10]Graduate School of Architecture, Planning and Preservation (GSAPP), Columbia University
[11]Data Science, Systems and Services Laboratory, ETH Zürich, Switzerland
[*]corresponding.mg2068@cam.ac.uk

S1 The Zollverein dataset

S1.1 The Zollverein wayfinding experiment in VR

S1.2 Dataset descriptive statistics

Column Name	Description
task	A semantically defined destination to be reached (i.e., roof-terrace, patio, office, auditiorium, reading area, study area)
task_order	The chronological order in which this task was executed
time_since_start_of_1st_trial	Elapsed time since the start of the first task (excludes registration and practice time)
total_distance	Distance walked by the participant
total_time	Total time taken to complete the task
building	Building condition in which this task was executed
age	Age of the participant
gender	Gender reported by the participant
average_speed	Average participant movement speed inside the VR environment
time_of_target_first_seen	Time since the start of the task when the target is first observed
total_distance_deviation	Total distance walked minus the distance of a straight line to the target
percentage_distance_deviation	Total distance deviation divide by he distance of a straight line to the target
vertical_head_movement	Average camera elevation change
horizontal_head_movement	Average camera azimuth change
visibility_sum	Sum of rays within the field of view that hit the target
visibility_avgerage	Rays within the field of view that hit the target averaged along the path
view_volume_sum	Sum of camera view volumes minus obstacles limiting this view along the path
view_volume_average	Camera view volume minus obstacles limiting this view volume averaged along the path
cosine_similarity_sum	Sum of cosines between the walking vectors at each step and the vector pointing to the final position
cosine_similarity_average	extttcosine_similarity_sum divided by the number of measurements
[after/before]_target_seen_[MEASURE]	[MEASURE] in the time window [after/before] the target has been seen
at_target_seen_view_volume	View volume at the moment the target is first seen
at_seen_[x,y,z]	[x,y,z] coordinate at the time the target was first seen
participant	Anonymized unique participant identifier
experimental_session	Instantiation of the experiment; data collection session
distance_inside	Walked distance inside the vertical circulation, stairs
distance_percentage_between_floors	distance_inside divided by total_distance
time_to_stairs	Time of the first arrival to the stairs
time_inside	Time the participant spent inside the vertical circulation, stairs
time_percentage_between_floors	time_inside divided by total_time

Table S1. *Overview of all columns in the dataset.* The dataset was generated on the basis of the VR experiment by[1]. This dataset contains 890 rows × 53 columns and is available at SSRN.

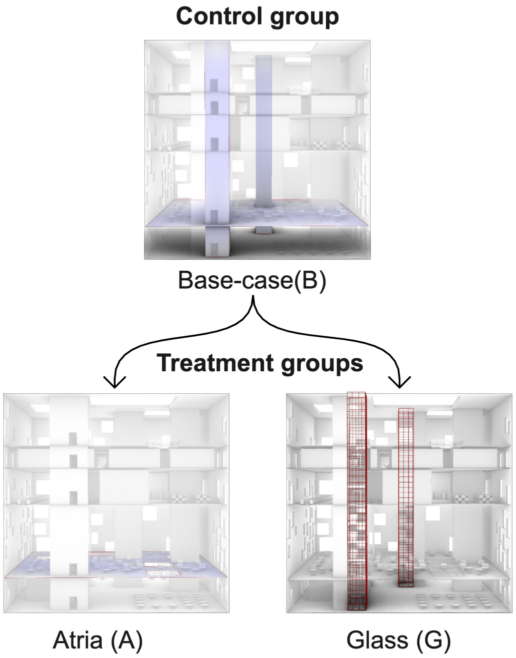

(a) For the VR study, a model of the Zollverein building was used as the Base-case building (top). This building was manipulated through changing the first floor or the staircase shafts. In the Atria condition, the floor of the first floor was interspersed with atria. In the Glass condition, the concrete shafts for the staircases were replaced with glass.

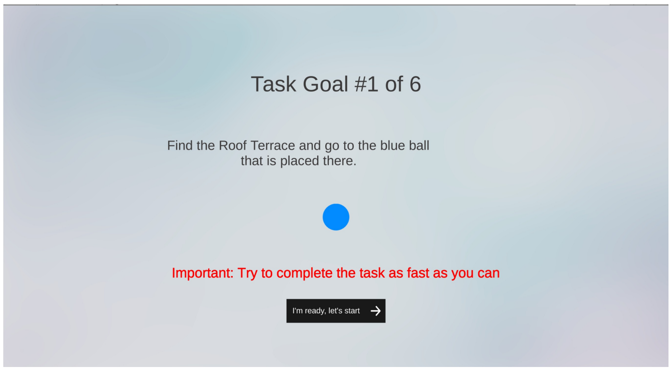

(b) An exemplary instruction screen provided to participants during the VR study. The instruction screen shows the progression through the tasks (counter at the top), the current instructions, the color associated with the goal (e.g. a blue sphere), and a hint to solve the task quickly. Participants can proceed at any time by clinging the button at the bottom.

Figure S1. *Details of the VR experimental design.* In Subfig. S1a, an overview of the building conditions is given. In Subfig. S1b, the instruction screen for the participants is shown.

The variables of interest for this study are reported in Tab. S3. A Tukey's range test[2] was used for a pairwise comparison for the ANOVA results to understand the difference between treatment groups. The mean age across conditions is very similar ($B = 33.75 \pm 8.07$ years, $A = 33.43 \pm 6.13$ years, $G = 33.80 \pm 6.07$ years) with a slightly stronger spread in age in the control group. The average speed across conditions appears very similar ($B = 1.17 \pm 0.13$ m/s, $A = 1.20 \pm 0.12$ m/s, $G = 1.12 \pm 0.20$ m/s) but the *Glass* treatment differs significantly from the others. The total distance moved per trial varies more visibly per condition ($B = 168.72 \pm 191.19$ m, $A = 154.85 \pm 144.16$ m, $G = 104.31 \pm 114.92$ m) with *Glass* treatment resulting in the significantly shorter time spend per trial than the other conditions. The total time spend per trial appears more similiar per condition ($B = 149.27 \pm 177.11$ s, $A = 131.41 \pm 124.04$ s, $G = 105.2 \pm 161.99$ s) with *Glass* treatment only resulting in a significantly shorter time spend per trial than the control group. The average view volume varies very strongly across treatments ($B = 1060.18 \pm 476.36$ m^2, $A = 1196.95 \pm 545.96$ m^2, $G = 1583.87 \pm 362.54$ m^2) with all conditions differing significantly from each other and the *Glass*condition resulting in the highest mean view volume and the lowest variation across trials. The percentage of the trajectories between floors is very similar between the control and the Atria treatment but significantly differs for the *Glass* treatment ($B = 29.63 \pm 23.43$ %, $A = 28.20 \pm 22.28$ %, $G = 42.29 \pm 29.45$ %). Lastly, the time participants take to move up from the ground floor to the first is significantly shorter in the *Glass*condition ($B = 25.45 \pm 30.43$ s, $A = 24.13 \pm 28.12$ s, $G = 14.86 \pm 18.27$ s).

	Trials				Participants			
	Base	Atria	Glass	Total	Base	Atria	Glass	Total
Male	161	240	226	627	27	40	38	105
Female	125	54	84	213	21	9	15	44
Total	286	294	310	890	48	49	53	149

Table S2. *Counts of trials and participants.* Trials and participants are ordered by treatment and gender distribution.

Variable	Building	Descriptive							Pairwise comparison Adjusted p-value†		
		Mean	Std.	Min.	25%	50%	75%	Max.	Base	Atria	Glass
Age	Base	33.75	8.07	18	29	33	37	59	-	0.83	1.00
	Atria	33.43	6.13	24	29	32	37	45	0.83	-	0.78
	Glass	33.80	6.07	21	29	33	39	46	1.00	0.78	-
Avg. speed	Base	1.17	0.13	0.42	1.13	1.21	1.27	1.31	-	0.16	**<0.05***
	Atria	1.20	0.12	0.39	1.16	1.24	1.27	1.31	0.16	-	**<0.05***
	Glass	1.12	0.20	0.32	1.07	1.18	1.26	1.30	**<0.05***	**<0.05***	-
Total distance	Base	168.72	191.19	16.58	41.088	120.28	218.06	1661.6	-	0.52	**<0.05***
	Atria	154.85	144.16	16.52	38.41	120.55	215.10	930.14	0.52	-	**<0.05***
	Glass	104.31	114.92	16.26	45.50	88.80	117.68	1093.54	**<0.05***	**<0.05***	-
Total time	Base	149.27	177.11	12.87	34.93	103.94	184.84	1477.42	-	0.35	**<0.05***
	Atria	131.41	124.04	13.00	32.87	95.80	190.30	787.32	0.35	-	0.10
	Glass	105.22	161.99	12.47	38.30	76.15	113.82	2161.10	**<0.05***	0.10	-
Avg. view volume	Base	1060.18	476.36	161.40	751.54	1084.66	1285.26	2284.16	-	**<0.05***	**<0.05***
	Atria	1196.95	545.96	159.01	829.56	1179.38	1395.97	2545.45	**<0.05***	-	**<0.05***
	Glass	1583.87	362.54	307.25	1341.79	1622.68	1820.95	3305.49	**<0.05***	**<0.05***	-
Percentage between floor	Base	29.63%	23.43%	0.00%	0.00%	31.44%	43.60%	81.67%	-	0.77	**<0.05***
	Atria	28.20%	22.28%	0.00%	0.00%	29.88%	40.92%	79.23%	0.77	-	**<0.05***
	Glass	42.29%	29.45%	0.00%	18.64%	40.78%	72.23%	88.41%	**<0.05***	**<0.05***	-
Time to move up	Base	25.45	30.43	0.00	0.00	16.11	35.12	165.58	-	0.81	**<0.05***
	Atria	24.13	28.12	0.00	0.00	21.00	32.56	246.25	0.81	-	**<0.05***
	Glass	14.86	18.27	0.00	6.46	8.94	17.65	138.82	**<0.05***	**<0.05***	-

Note: †:A Tukey's range test was used to perform a pairwise comparison for ANOVA[2].

Table S3. *Distribution of variables.* Distributions of variables used in this study from the summary dataset[1].

	Task	Task Order	TimeSince Startof 1stTrial	Dist	Tot Time	Building	Age	Gender	Avg Speed	First Seen	Total Distance Deviation	Percentual Distance Deviation	Vertical Head Movement	...	Participant
**0**	5.0	2.0	256.7	122.6	104.6	2.0	33.0	0.0	1.2	90.8	98.8	4.1	0.2	...	613ded062c9c429e2ec108cbb3172098
**1**	3.0	4.0	326.2	61.0	50.1	2.0	33.0	0.0	1.2	0.0	30.2	1.0	0.1	...	613ded062c9c429e2ec108cbb3172098
**2**	1.0	3.0	273.2	17.6	13.8	2.0	33.0	0.0	1.3	0.0	2.1	0.1	0.1	...	613ded062c9c429e2ec108cbb3172098
**3**	6.0	5.0	424.2	107.2	94.2	2.0	33.0	0.0	1.1	76.3	78.7	2.8	0.2	...	613ded062c9c429e2ec108cbb3172098
**...**	...	...	...	...	...	...	...	...	...	...	...	...	...	...	...
**887**	4.0	5.0	455.4	172.0	135.8	2.0	21.0	0.0	1.3	108.5	148.3	6.2	0.0	...	acdd303a2f1eb99eab706b71186224d6
**888**	5.0	1.0	228.6	115.1	91.5	2.0	21.0	0.0	1.3	77.1	91.3	3.8	0.0	...	acdd303a2f1eb99eab706b71186224d6
**889**	6.0	0.0	132.3	167.9	132.3	2.0	21.0	0.0	1.3	102.3	137.5	4.5	0.0	...	acdd303a2f1eb99eab706b71186224d6

Table S4. *Preview of data.* The format of the Zollverein summary data set[1] is shown. Here, each row corresponds to a single wayfinding task performed by one participant in one of the three building conditions.

S2 Model Comparisons

To compare our models we apply anovas to identify with models fit best. First, we compare with linear models in Tab. S7. We observe that LMER significantly performs better than the linear model for every variable except ‘Distance’. Furthermore, we note that the linear model with covariates does not improve the model quality significantly. Second, we compare the LMER models to discern whether covariates improve our models in Tab. S8. We observe that for velocity and the ‘time to move up’, the models with covariates significantly improve the model fit.

To overcome interpretability issues of complex models, we opt to represent results as marginal effects which are ill-defined[3] and therefore need to be clearly defined and used acknowledging known biases to be used for triangulation[4]. We opt for Average Marginal Effects (AME)[5] and Marginal Effects at the Mean (MEM)[6] because both are common in different disciplines. MEM is simpler as predicted values for task and visibility treatment are compared to the average response. However, it is noted that the average respond may not exist in real data[7] and is therefore rather abstract in its implication to the real world. AME tries to improve this by calculating a model prediction for all real inputs and averaging over task and visibility treatment. We compare AME and MEM for our wayfinding efficiency measures in Fig. S2 and our wayfinding strategy measures in Fig. S3. First, we notice that the overall pattern between visibility treatments remains visually similar across AME and MEM. However, there is a slight difference in values that is visually notable for time and velocity. Nonetheless, the type of marginal effects does not impact the overall outcome. Lastly, for the models with a significant improved fit with covariates, we investigate the marginal effects and observe, that only for velocity under AME we find a large substantial impact of covariates.

Finally, we are comparing our triangulation measures to determine whether our effects are robust to differences in measurement. First, for wayfinding efficiency, we find that distance and total time have very similar patterns at different scales. Velocity has a different pattern but also shows that glass is different. Distance produces the substantially largest difference between treatment conditions and is selected for the main text. Second, for wayfinding strategy, we find all measures produce a similar pattern (with Time to move up being on an inverted scale). The ratio measure has a lower response strength for one task (Reading Area) but stronger responses for other tasks (Office, Patio, and Roof Terrace). We find that the Percentage measure has the smallest confidence intervals and select it for the main text. We believe that across measures and methods we can show robust results for our main claims.

For wayfinding efficiency, we use distance (Eq. **??**), time (Eq. S1), and average velocity (Eq. S2) as three distinct triangulating[4] measures. For wayfinding strategy, we use the percentage of time spend between floors (Eq. **??**), the ratio of distance between floors and within floors (Eq. S3), and the time to move up (Eq. S4) the first floor[1] as three distinct triangulating[4] measures. Furthermore, we compare linear models and LMER and check for the impact of gender and age, see Tab. S5 for wayfinding efficiency and Tab. S6 for wayfinding strategy.

$$\text{Total Time} \sim \text{Visibility Treatment} * \text{Task} + Age + Gender + (1|\text{Participant}) \tag{S1}$$

$$\text{Average Velocity} \sim \text{Visibility Treatment} * \text{Task} + Age + Gender + (1|\text{Participant}) \tag{S2}$$

$$\text{Ratio Between/Within Floor} \sim \text{Visibility Treatment} * \text{Task} + Age + Gender + (1|\text{Participant}) \tag{S3}$$

$$\text{Time To Move Up} \sim \text{Visibility Treatment} * \text{Task} + Age + Gender + (1|\text{Participant}) \tag{S4}$$

[1]For each trial, the time taken to reach from the same starting position to the first step of the staircase in the ground floor (in either of the two circulation cores) was calculated by evaluating participants’ camera height (set by default to 1.7 meters). ‘Time to Move Up’ is the point in time when participants’ camera’s height exceeds 2 meters, (i.e. the first step of the stairs).

Models for Wayfinding Efficency

	Distance				Total Time				Average Speed			
	Without Covariates		With Covariates		Without Covariates		With Covariates		Without Covariates		With Covariates	
	LMER	*LM*	*LMER*	*LM*	*LMER*	*LM*	*LMER*	*LM*	*LMER*	*LM*	*LMER*	*LM*
**Treatments and Tasks**												
Base+Auditorium	62.832***	62.832***	50.539	50.489*	56.633***	56.633***	22.946	22.809	1.159***	1.159***	1.310***	1.311***
(Constant)	(19.156)	(19.155)	(30.868)	(29.526)	(20.361)	(20.356)	(35.969)	(31.279)	(0.022)	(0.022)	(0.054)	(0.033)
Atrium	−1.472	−1.472	2.050	2.084	−3.419	−3.419	3.589	3.674	−0.014	−0.014	−0.040	−0.040
	(26.952)	(26.951)	(27.096)	(27.071)	(28.648)	(28.641)	(28.774)	(28.678)	(0.031)	(0.031)	(0.030)	(0.030)
Glass	−39.909	−39.909	−37.613	−37.590	−35.456	−35.456	−30.944	−30.887	−0.031	−0.031	−0.047	−0.047
	(26.565)	(26.563)	(26.632)	(26.617)	(28.236)	(28.229)	(28.253)	(28.197)	(0.031)	(0.031)	(0.030)	(0.029)
Reading Area	18.777	18.777	18.777	18.777	14.190	14.190	14.190	14.190	0.018	0.018	0.018	0.018
	(26.655)	(27.089)	(26.658)	(27.089)	(27.080)	(28.788)	(27.083)	(28.698)	(0.019)	(0.032)	(0.019)	(0.030)
Study Area	130.009***	130.009***	130.009***	130.009***	110.056***	110.056***	110.056***	110.056***	0.055***	0.055*	0.055***	0.055*
	(26.655)	(27.089)	(26.658)	(27.089)	(27.080)	(28.788)	(27.083)	(28.698)	(0.019)	(0.032)	(0.019)	(0.030)
Office	193.299***	192.439***	193.354***	192.498***	164.282***	162.175***	164.316***	162.325***	0.020	0.022	0.020	0.021
	(26.952)	(27.382)	(26.955)	(27.382)	(27.396)	(29.100)	(27.398)	(29.008)	(0.019)	(0.032)	(0.019)	(0.030)
Patio	190.921***	190.921***	190.921***	190.921***	173.415***	173.415***	173.415***	173.415***	−0.004	−0.004	−0.004	−0.004
	(26.655)	(27.089)	(26.658)	(27.089)	(27.080)	(28.788)	(27.083)	(28.698)	(0.019)	(0.032)	(0.019)	(0.030)
Roof Terrace	106.764***	106.764***	106.764***	106.764***	98.909***	98.909***	98.909***	98.909***	−0.007	−0.007	−0.007	−0.007
	(26.655)	(27.089)	(26.658)	(27.089)	(27.080)	(28.788)	(27.083)	(28.698)	(0.019)	(0.032)	(0.019)	(0.030)
**Interaction Effects Between Treatments and Tasks**												
Atrium*Reading Area	−7.531	−7.531	−7.531	−7.531	−6.143	−6.143	−6.143	−6.143	0.048*	0.048	0.048*	0.048
	(37.503)	(38.114)	(37.507)	(38.114)	(38.101)	(40.505)	(38.105)	(40.377)	(0.027)	(0.045)	(0.027)	(0.042)
Atrium*Study Area	−114.618***	−114.618***	−114.618***	−114.618***	−100.610***	−100.610**	−100.610***	−100.610**	0.034	0.034	0.034	0.034
	(37.503)	(38.114)	(37.507)	(38.114)	(38.101)	(40.505)	(38.105)	(40.377)	(0.027)	(0.045)	(0.027)	(0.042)
Atrium*Office	−7.633	−6.773	−7.688	−6.833	−11.484	−9.376	−11.517	−9.527	0.050*	0.049	0.050*	0.050
	(37.715)	(38.323)	(37.719)	(38.323)	(38.326)	(40.726)	(38.330)	(40.598)	(0.027)	(0.045)	(0.027)	(0.042)
Atrium*Patio	66.585*	66.585*	66.585*	66.585*	43.284	43.284	43.284	43.284	0.059**	0.059	0.059**	0.059
	(37.503)	(38.114)	(37.507)	(38.114)	(38.101)	(40.505)	(38.105)	(40.377)	(0.027)	(0.045)	(0.027)	(0.042)
Atrium*Roof Terrace	−15.636	−15.636	−15.636	−15.636	−16.730	−16.730	−16.730	−16.730	0.039	0.039	0.039	0.039
	(37.503)	(38.114)	(37.507)	(38.114)	(38.101)	(40.505)	(38.105)	(40.377)	(0.027)	(0.045)	(0.027)	(0.042)
Glass*Reading Area	18.459	18.459	18.459	18.459	20.519	20.519	20.519	20.519	−0.019	−0.019	−0.019	−0.019
	(36.964)	(37.566)	(36.968)	(37.566)	(37.553)	(39.922)	(37.558)	(39.796)	(0.026)	(0.044)	(0.026)	(0.042)
Glass*Study Area	−56.186	−56.186	−56.186	−56.186	−39.810	−39.810	−39.810	−39.810	−0.032	−0.032	−0.032	−0.032
	(36.964)	(37.566)	(36.968)	(37.566)	(37.553)	(39.922)	(37.558)	(39.796)	(0.026)	(0.044)	(0.026)	(0.042)
Glass*Office	−60.572	−59.742	−60.715	−59.903	−35.811	−33.734	−36.006	−34.127	−0.031	−0.031	−0.031	−0.029
	(37.267)	(37.865)	(37.272)	(37.866)	(37.876)	(40.241)	(37.881)	(40.114)	(0.026)	(0.044)	(0.026)	(0.042)
Glass*Patio	−45.475	−45.475	−45.475	−45.475	−9.278	−9.278	−9.278	−9.278	−0.028	−0.028	−0.028	−0.028
	(36.964)	(37.566)	(36.968)	(37.566)	(37.553)	(39.922)	(37.558)	(39.796)	(0.026)	(0.044)	(0.026)	(0.042)
Glass*Roof Terrace	−6.279	−6.310	−6.368	−6.412	9.141	9.110	8.980	8.869	−0.032	−0.030	−0.031	−0.029
	(37.054)	(37.654)	(37.058)	(37.655)	(37.649)	(40.016)	(37.653)	(39.890)	(0.026)	(0.044)	(0.026)	(0.042)
**Covariates**												
Age			0.188	0.187			0.651	0.651			−0.003**	−0.003***
			(0.707)	(0.656)			(0.867)	(0.695)			(0.001)	(0.001)
Gender			13.619	13.755			26.732**	27.068**			−0.097***	−0.097***
			(10.789)	(10.016)			(13.234)	(10.611)			(0.022)	(0.011)
Observations	890	890	890	890	890	890	890	890	890	890	890	890
Adjusted R^2		0.264		0.264		0.191		0.196		0.047		0.143
Log Likelihood	−5,533.919		−5,529.208		−5,576.701		−5,570.071		623.670		626.537	
Akaike Inf. Crit.	11,107.840		11,102.420		11,193.400		11,184.140		−1,207.339		−1,209.074	
Bayesian Inf. Crit.	11,203.660		11,207.820		11,289.230		11,289.550		−1,111.515		−1,103.667	
Residual Std. Error		132.709 (df = 872)		132.710 (df = 870)		141.034 (df = 872)		140.589 (df = 870)		0.155 (df = 872)		0.147 (df = 870)
F Statistic		19.784*** (df = 17; 872)		17.806*** (df = 19; 870)		13.326*** (df = 17; 872)		12.395*** (df = 19; 870)		3.593*** (df = 17; 872)		8.811*** (df = 19; 870)

Note: *p<0.1; **p<0.05; ***p<0.01

Table S5. *Model Comparison of Wayfinding Efficiency.*

Models for Wayfinding Strategy

	Percentage of Time Spend Between Floors				Ratio of Distance Spend Between Floors and Within Floors				Time Spend Before Moving Up			
	Without Covariates		With Covariates		Without Covariates		With Covariates		Without Covariates		With Covariates	
	LMER	*LM*	*LMER*	*LM*	*LMER*	*LM*	*LMER*	*LM*	*LMER*	*LM*	*LMER*	*LM*
**Treatments and Tasks**												
Base+Auditorium	0.045**	0.045**	0.075**	0.076***	0.088	0.088	0.178	0.179	1.013	1.013	−10.475**	−10.509**
(Constant)	(0.018)	(0.018)	(0.031)	(0.028)	(0.115)	(0.115)	(0.191)	(0.178)	(3.123)	(3.123)	(5.204)	(4.786)
Atrium	0.006	0.006	0.007	0.007	0.0001	0.0001	0.004	0.004	−0.059	−0.059	0.839	0.843
	(0.026)	(0.026)	(0.026)	(0.026)	(0.162)	(0.162)	(0.163)	(0.163)	(4.394)	(4.394)	(4.396)	(4.388)
Glass	0.014	0.014	0.015	0.015	−0.011	−0.011	−0.008	−0.008	1.069	1.069	1.602	1.605
	(0.025)	(0.025)	(0.025)	(0.025)	(0.160)	(0.160)	(0.160)	(0.160)	(4.330)	(4.330)	(4.319)	(4.314)
Reading Area	0.224***	0.224***	0.224***	0.224***	0.291*	0.291*	0.291*	0.291*	35.354***	35.354***	35.354***	35.354***
	(0.025)	(0.026)	(0.025)	(0.026)	(0.159)	(0.163)	(0.159)	(0.163)	(4.252)	(4.416)	(4.252)	(4.391)
Study Area	0.019	0.019	0.019	0.019	0.014	0.014	0.014	0.014	4.432	4.432	4.432	4.432
	(0.025)	(0.026)	(0.025)	(0.026)	(0.159)	(0.163)	(0.159)	(0.163)	(4.252)	(4.416)	(4.252)	(4.391)
Office	0.328***	0.328***	0.327***	0.328***	0.557***	0.560***	0.557***	0.560***	32.944***	32.791***	32.960***	32.836***
	(0.025)	(0.026)	(0.025)	(0.026)	(0.160)	(0.165)	(0.160)	(0.165)	(4.300)	(4.464)	(4.301)	(4.438)
Patio	0.356***	0.356***	0.356***	0.356***	0.657***	0.657***	0.657***	0.657***	50.166***	50.166***	50.166***	50.166***
	(0.025)	(0.026)	(0.025)	(0.026)	(0.159)	(0.163)	(0.159)	(0.163)	(4.252)	(4.416)	(4.252)	(4.391)
Roof Terrace	0.581***	0.581***	0.581***	0.581***	2.083***	2.083***	2.083***	2.083***	24.246***	24.246***	24.246***	24.246***
	(0.025)	(0.026)	(0.025)	(0.026)	(0.159)	(0.163)	(0.159)	(0.163)	(4.252)	(4.416)	(4.252)	(4.391)
**Interaction Effects Between Treatments and Tasks**												
Atrium*Reading Area	0.012	0.012	0.012	0.012	0.047	0.047	0.047	0.047	−6.495	−6.495	−6.495	−6.495
	(0.034)	(0.036)	(0.034)	(0.036)	(0.223)	(0.229)	(0.223)	(0.229)	(5.982)	(6.213)	(5.983)	(6.178)
Atrium*Study Area	−0.019	−0.019	−0.019	−0.019	−0.016	−0.016	−0.016	−0.016	−2.092	−2.092	−2.092	−2.092
	(0.034)	(0.036)	(0.034)	(0.036)	(0.223)	(0.229)	(0.223)	(0.229)	(5.982)	(6.213)	(5.983)	(6.178)
Atrium*Office	−0.042	−0.043	−0.042	−0.043	−0.112	−0.115	−0.112	−0.115	0.098	0.250	0.082	0.206
	(0.035)	(0.036)	(0.035)	(0.036)	(0.224)	(0.230)	(0.224)	(0.230)	(6.017)	(6.247)	(6.017)	(6.212)
Atrium*Patio	−0.053	−0.053	−0.053	−0.053	−0.117	−0.117	−0.117	−0.117	−7.301	−7.301	−7.301	−7.301
	(0.034)	(0.036)	(0.034)	(0.036)	(0.223)	(0.229)	(0.223)	(0.229)	(5.982)	(6.213)	(5.983)	(6.178)
Atrium*Roof Terrace	−0.020	−0.020	−0.020	−0.020	−0.275	−0.275	−0.275	−0.275	7.676	7.676	7.676	7.676
	(0.034)	(0.036)	(0.034)	(0.036)	(0.223)	(0.229)	(0.223)	(0.229)	(5.982)	(6.213)	(5.983)	(6.178)
Glass*Reading Area	0.088***	0.088**	0.088***	0.088**	0.233	0.233	0.233	0.233	−19.416***	−19.416***	−19.416***	−19.416***
	(0.034)	(0.036)	(0.034)	(0.036)	(0.220)	(0.226)	(0.220)	(0.226)	(5.896)	(6.124)	(5.897)	(6.089)
Glass*Study Area	0.061*	0.061*	0.061*	0.061*	0.111	0.111	0.111	0.111	−1.610	−1.610	−1.610	−1.610
	(0.034)	(0.036)	(0.034)	(0.036)	(0.220)	(0.226)	(0.220)	(0.226)	(5.896)	(6.124)	(5.897)	(6.089)
Glass*Office	0.232***	0.233***	0.233***	0.233***	2.160***	2.159***	2.161***	2.160***	−14.996**	−14.895**	−15.067**	−15.001**
	(0.034)	(0.036)	(0.034)	(0.036)	(0.222)	(0.228)	(0.222)	(0.228)	(5.946)	(6.173)	(5.946)	(6.138)
Glass*Patio	0.183***	0.183***	0.183***	0.183***	1.295***	1.295***	1.295***	1.295***	−23.683***	−23.683***	−23.683***	−23.683***
	(0.034)	(0.036)	(0.034)	(0.036)	(0.220)	(0.226)	(0.220)	(0.226)	(5.896)	(6.124)	(5.897)	(6.089)
Glass*Roof Terrace	0.117***	0.118***	0.117***	0.118***	1.476***	1.478***	1.476***	1.478***	−10.549*	−10.600*	−10.604*	−10.662*
	(0.034)	(0.036)	(0.034)	(0.036)	(0.221)	(0.226)	(0.221)	(0.226)	(5.911)	(6.138)	(5.911)	(6.103)
**Covariates**												
Age			−0.001	−0.001			−0.003	−0.003			0.300**	0.300***
			(0.001)	(0.001)			(0.004)	(0.004)			(0.122)	(0.106)
Gender			0.008	0.008			0.019	0.019			3.131*	3.146*
			(0.011)	(0.009)			(0.068)	(0.060)			(1.861)	(1.624)
Observations	890	890	890	890	890	890	890	890	890	890	890	890
Adjusted R^2		0.768		0.768		0.633		0.632		0.328		0.335
Log Likelihood	542.307		533.536		−1,072.710		−1,078.737		−3,948.276		−3,943.455	
Akaike Inf. Crit.	−1,044.613		−1,023.072		2,185.420		2,201.473		7,936.551		7,930.909	
Bayesian Inf. Crit.	−948.789		−917.665		2,281.245		2,306.880		8,032.376		8,036.316	
Residual Std. Error		0.126 (df = 872)		0.126 (df = 870)		0.798 (df = 872)		0.798 (df = 870)		21.634 (df = 872)		21.511 (df = 870)
F Statistic		174.034*** (df = 17; 872)		156.085*** (df = 19; 870)		91.129*** (df = 17; 872)		81.443*** (df = 19; 870)		26.482*** (df = 17; 872)		24.602*** (df = 19; 870)
Note:												*p<0.1; **p<0.05; ***p<0.01

Table S6. *Model Comparison of Wayfinding Strategy.*

Comparing model fit

Variable	Model	Covariates	#Params	AIC	BIC	logLik	deviance	χ^2	Df	Pr(> χ^2)
Time	LM	Without	19	11354.74	11445.78	-5658.37	11316.74			
		With	21	11351.08	11451.70	-5654.54	11309.08	0.00	1	1.00
	LMER	Without	20	11333.79	11429.62	-5646.90	11293.79	22.95	1	0.00
		With	22	11332.98	11438.38	-5644.49	11288.98	20.10	1	0.00
Distance	LM	Without	19	11246.45	11337.48	-5604.23	11208.45			
		With	21	11248.42	11349.03	-5603.21	11206.42	0.05	1	0.83
	LMER	Without	20	11246.47	11342.29	-5603.23	11206.47	1.99	1	0.16
		With	22	11248.73	11354.14	-5602.37	11204.73	1.68	1	0.19
Velocity	LM	Without	19	-773.01	-681.98	405.51	-811.01			
		With	21	-865.38	-764.77	453.69	-907.38	0.00	1	1.00
	LMER	Without	20	-1322.94	-1227.12	681.47	-1362.94	551.93	1	0.00
		With	22	-1342.15	-1236.74	693.07	-1386.15	478.76	1	0.00
Percentage	LM	Without	19	-1144.49	-1053.46	591.25	-1182.49			
		With	21	-1143.71	-1043.09	592.85	-1185.71	0.00	1	1.00
	LMER	Without	20	-1156.84	-1061.02	598.42	-1196.84	14.35	1	0.00
		With	22	-1155.03	-1049.62	599.51	-1199.03	13.32	1	0.00
Ratio	LM	Without	19	2142.92	2233.95	-1052.46	2104.92			
		With	21	2146.27	2246.88	-1052.13	2104.27	0.00	1	1.00
	LMER	Without	20	2139.87	2235.69	-1049.93	2099.87	5.05	1	0.02
		With	22	2143.35	2248.76	-1049.68	2099.35	4.91	1	0.03
Time to move up	LM	Without	19	8017.75	8108.78	-3989.88	7979.75			
		With	21	8009.51	8110.13	-3983.76	7967.51	2.20	1	0.14
	LMER	Without	20	8009.71	8105.54	-3984.86	7969.71	10.04	1	0.00
		With	22	8004.58	8109.99	-3980.29	7960.58	6.93	1	0.01

Table S7. *ANOVA with linear model as base line.*

Variable	Model	Covariates	#Params	AIC	BIC	logLik	deviance	χ^2	Df	Pr(> χ^2)
Time	LMER	Without	20	11333.79	11429.62	-5646.90	11293.79			
		With	22	11332.98	11438.38	-5644.49	11288.98	4.82	2	0.09
Distance	LMER	Without	20	11246.47	11342.29	-5603.23	11206.47			
		With	22	11248.73	11354.14	-5602.37	11204.73	1.73	2	0.42
Velocity	LMER	Without	20	-1322.94	-1227.12	681.47	-1362.94			
		With	22	-1342.15	-1236.74	693.07	-1386.15	23.20	2	0.00
Percentage	LMER	Without	20	-1156.84	-1061.02	598.42	-1196.84			
		With	22	-1155.03	-1049.62	599.51	-1199.03	2.19	2	0.33
Ratio	LMER	Without	20	2139.87	2235.69	-1049.93	2099.87			
		With	22	2143.35	2248.76	-1049.68	2099.35	0.52	2	0.77
Time to move up	LMER	Without	20	8009.71	8105.54	-3984.86	7969.71			
		With	22	8004.58	8109.99	-3980.29	7960.58	9.13	2	0.01

Table S8. *ANOVA with LMER as base line.*

Marginal Effects for Wayfinding Efficiency

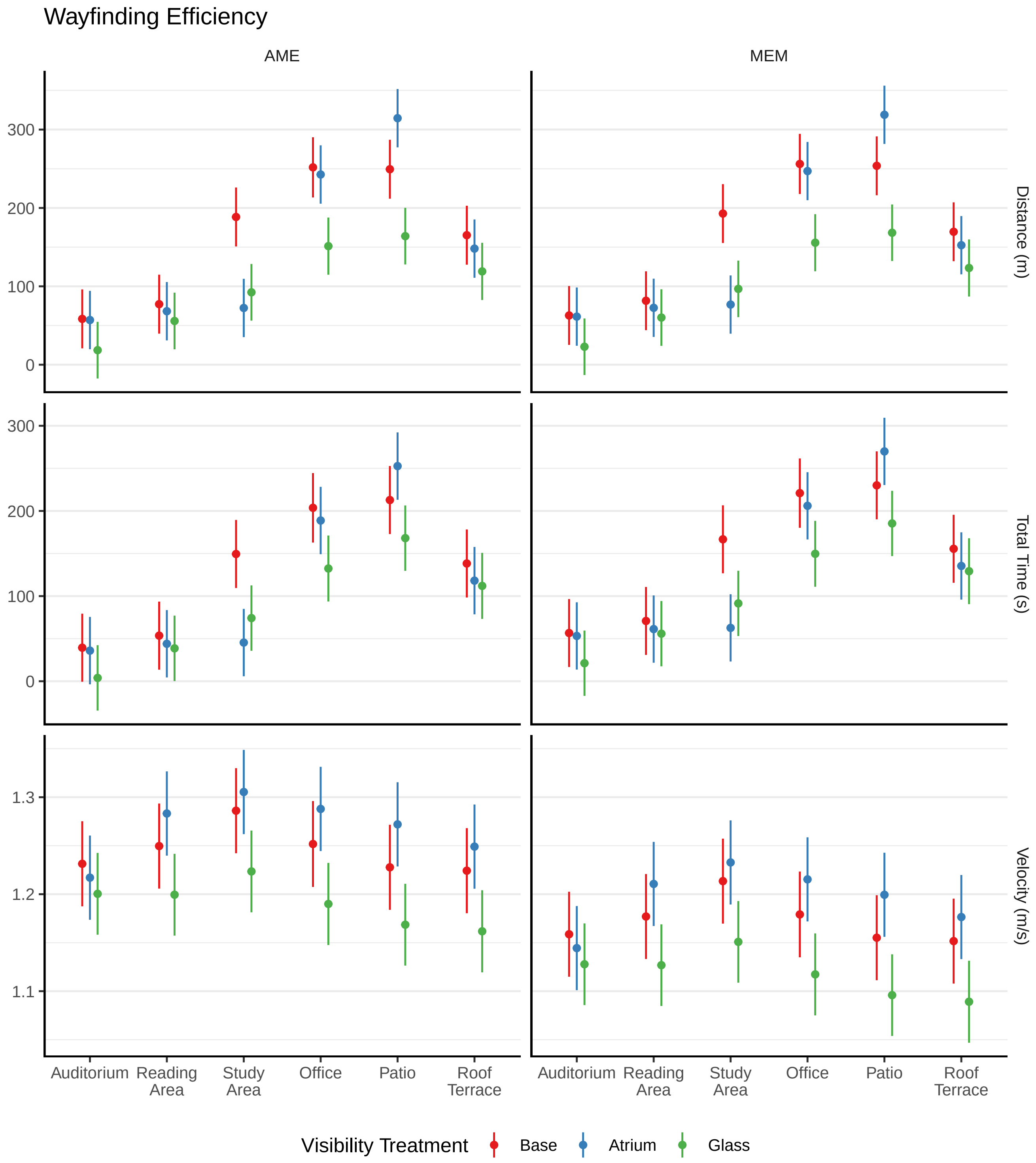

Figure S2. *Comparison of measurements of marginal effects of visibility and tasks on wayfinding efficiency.* Average Marginal Effects (AME)[5] and Marginal Effects at the Mean (MEM)[6] are calculated to robustly show the model outcomes.

Marginal Effects for Wayfinding Strategy

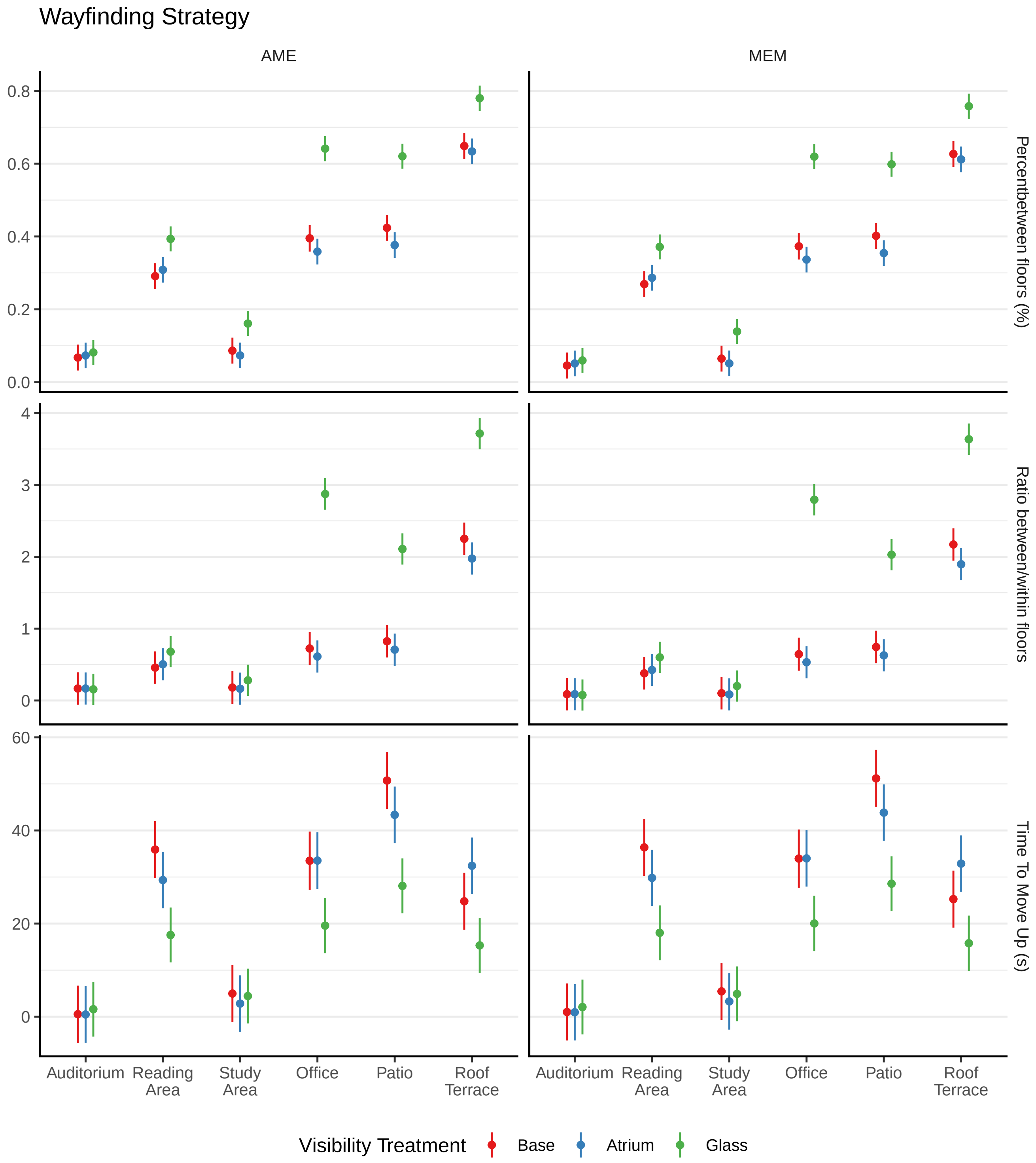

Figure S3. *The effect of visibility and tasks on wayfinding efficiency and strategy.* Average Marginal Effects (AME)[5] and Marginal Effects at the Mean (MEM)[6] are calculated to robustly show the model outcomes.

Marginal Effects for covariates

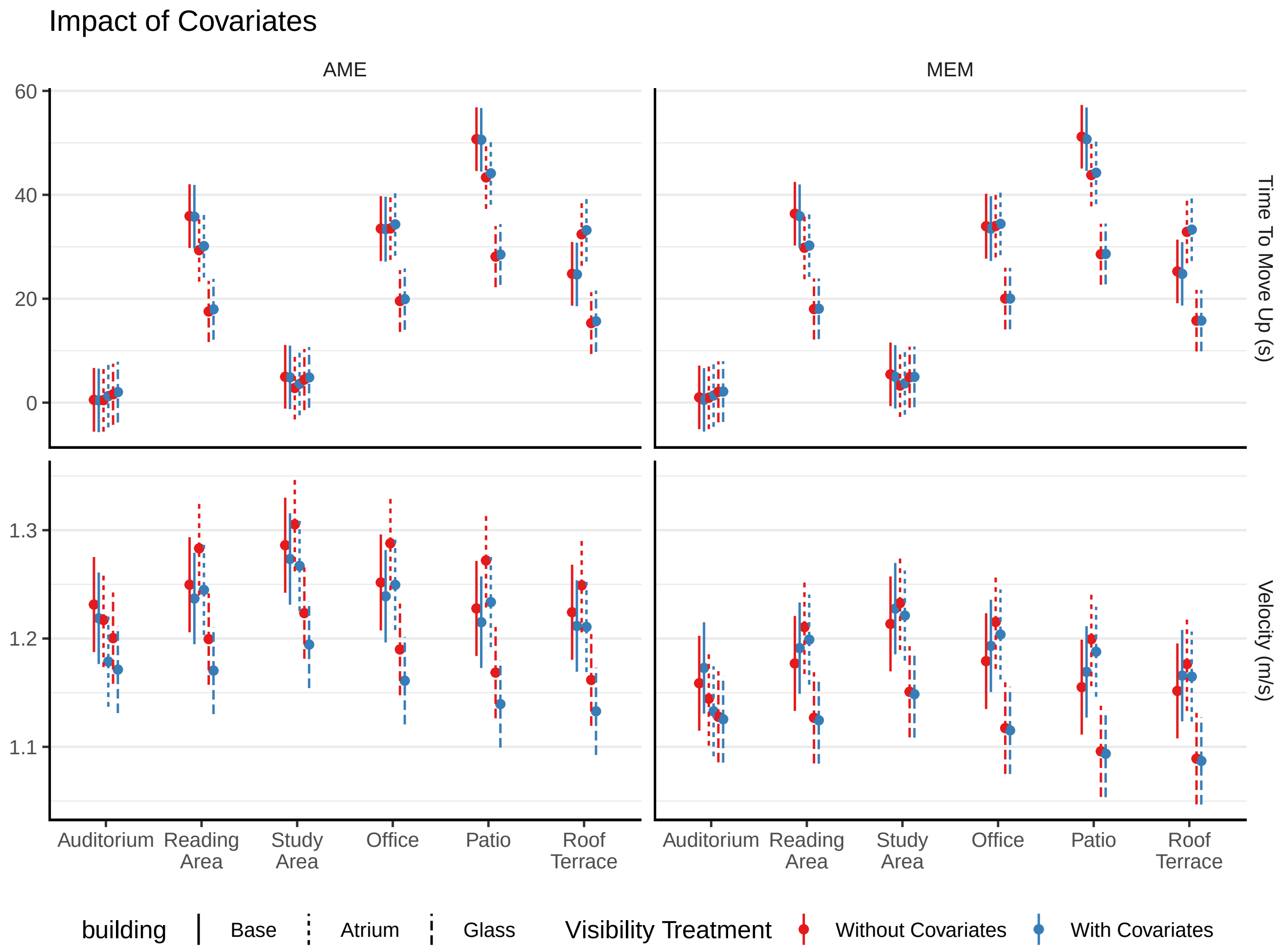

Figure S4. *The effect of covariates for visibility and tasks on selected wayfinding efficiency and strategy.* Average Marginal Effects (AME)[5] and Marginal Effects at the Mean (MEM)[6] are calculated to robustly show the model outcomes. 'Time to move up' is not substantially affected by covariates. Velocity is only substantially affect under AME.

S3 Non-parametric KDE Test

The two-sample comparisons of multivariate data can be evaluated with non-parametric test on a KDE[8]. The non-parametric density estimation is obtained by kernel smoothing due to its intuitive construction and interpretation[9]. Based on the statistical framework for common densities[10], we define f_1 and f_2 as d-variate random samples $X_1, X_2, \ldots, X_{n_1}$ and $Y_1, Y_2, \ldots, Y_{n_2}$. For a Kernel K,see Eq. S7, the density estimates of f_1 and f_2 can be constructed as in Eq. S5 and S6 where H_l is a bandwidth matrix for $l = 1, 2$.

$$\hat{f}_1(\mathbf{x}, \mathbf{H_1}) = \frac{1}{n_1} \sum_{i=1}^{n_1} K_{\mathbf{H_1}}(\mathbf{x} - \mathbf{X_i}) \tag{S5}$$

$$\hat{f}_2(\mathbf{x}, \mathbf{H_2}) = \frac{1}{n_2} \sum_{i=}^{n_2} K_{\mathbf{H_2}}(\mathbf{x} - \mathbf{Y_i}) \tag{S6}$$

$$K_{\mathbf{H_l}}(\mathbf{x}) = |\mathbf{H_l}|^{-\frac{1}{2}} K(\mathbf{H_l}^{-\frac{1}{2}} \mathbf{x}) \tag{S7}$$

To test the null hypothesis $H_0 : f_1 = f_2$ a test statistic is derived from a discrepancy measure[11], shown in Eq. S8, and can be rewritten in terms of the density estimates[8], shown in Eq. S9 where $\psi_l = \int f_l(\mathbf{x})^2 d\mathbf{x}$ for $l = 1, 2$ and $\psi_{i,j} = \int f_i(\mathbf{x}) f_j(\mathbf{x}) d\mathbf{x}$.

$$T = \int [f_1(\mathbf{x}) - f_2(\mathbf{x})]^2 d\mathbf{x} \tag{S8}$$

$$T = \psi_1 + \psi_2 - (\psi_{1,2} + \psi_{1,2}) \tag{S9}$$

The test statistic, shown in Eq. S10 is composed based on density estimates Eq. S5 and S6 and yields the terms shown in Eq. S11,S12,S13, and S14.

$$\hat{T} = \hat{\psi}_1 + \hat{\psi}_2 - (\hat{\psi}_{1,2} + \hat{\psi}_{2,1}) \tag{S10}$$

$$\hat{\psi}_1 = \frac{1}{n_1^2} \sum_{i_1=1}^{n_1} \sum_{i_2=1}^{n_1} K_{\mathbf{H_1}}(\mathbf{X_{i_1}} - \mathbf{X_{i_2}}) \tag{S11}$$

$$\hat{\psi}_2 = \frac{1}{n_2^2} \sum_{j_1=1}^{n_2} \sum_{j_2=1}^{n_2} K_{\mathbf{H_2}}(\mathbf{Y_{j_1}} - \mathbf{Y_{j_2}}) \tag{S12}$$

$$\hat{\psi}_{1,2} = \frac{1}{n_1 n_2} \sum_{i=1}^{n_1} \sum_{j=1}^{n_2} K_{\mathbf{H_1}}(\mathbf{X_i} - \mathbf{Y_j}) \tag{S13}$$

$$\hat{\psi}_{2,1} = \frac{1}{n_1 n 2} \sum_{i=1}^{n_1} \sum_{j=1}^{n_2} K_{\mathbf{H_2}}(\mathbf{X_i} - \mathbf{Y_j}) \tag{S14}$$

(S15)

The test statistic T describes the intra-sample pairwise differences (Eq. S11 and S12) to the inter-sample pairwise differences (Eq. S13 and S14) for the two distributions[8]. That is, if the inter-sample differences are larger than the intra-sample differences, the two distributions are different and H_0 is rejected.

S3.1 K-Means clustering of wayfinding paths

Number of Clusters (n)	Average Silhouette Score	Number of Samples/Participants in Each Cluster
2	0.80178624	[110, 36]
3	0.5610873	[71, 36, 39]
4	0.57777685	[52, 36, 36, 22]
5	0.5699246	[42, 36, 34, 22, 12]
6	0.58031696	[22, 36, 34, 22, 12, 20]

Table S9. Average Silhouette Scores and Number of Samples/Participants for Different Numbers of Clusters

S3.2 Staircase usage across building conditions

To investigate the preference for front and back staircases across different building conditions, we conducted an analysis of the percentage of distance participants spent using each staircase. The aim was to compare the utilization patterns of these staircases in the Glass Atria, and Base groups. Our findings are summarized in TableS10, which provides key statistics for each building condition:

From the table, we observe interesting trends. Participants in the *Glass* group overwhelmingly favored the front staircase, with a mean usage of 38.38%. In contrast, the *Atria* group also showed a preference for the front staircase (mean usage of 13.74%), but the difference from the back staircase was not statistically significant. Similarly, in the *Base* condition, the back staircase was utilized slightly more (mean usage of 11.49%) than the front staircase (mean usage of 18.19%), although this difference was not statistically significant.

In summary, our analysis reveals varying staircase usage preferences across building conditions. Participants in the *Glass* group exhibited a strong preference for the front staircase, while the *Atria* and*Base* conditions showed less distinct staircase usage patterns.

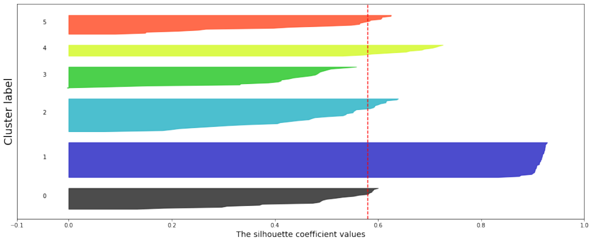

(a) 6 clusters

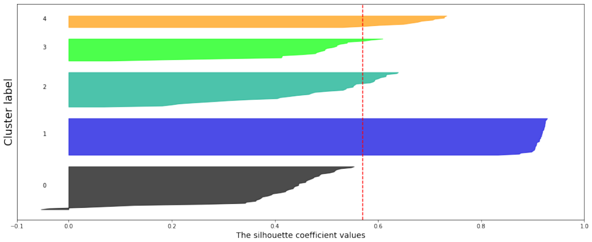

(b) 5 clusters

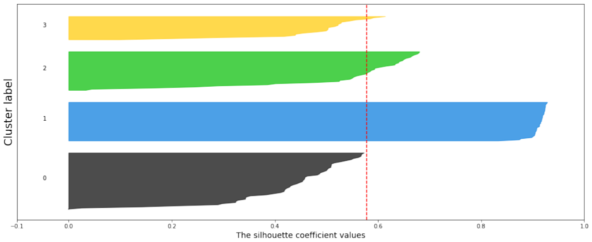

(c) 4 clusters

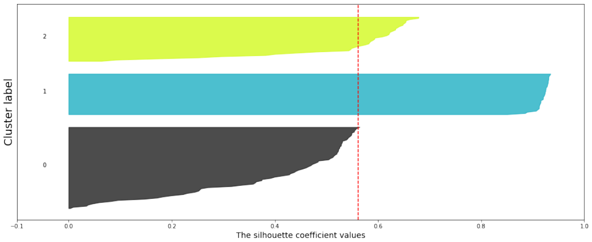

(d) 3 clusters

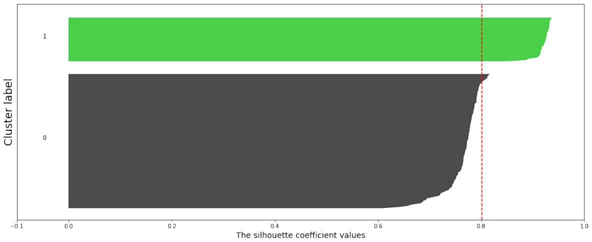

(e) 2 clusters

Figure S4. *Average Silhouette Scores for Different Numbers of Clusters* Silhouette scores were calculated for varying numbers of clusters ($n_{\text{clusters}} = 2, 3, 4, 5$, and 6). The silhouette scores indicate the degree of similarity of data points within their own clusters compared to neighboring clusters. As the number of clusters increases, the average silhouette scores change accordingly: for $n_{\text{clusters}} = 2$, the score is 0.8018 with 110 persons in cluster 0 and 36 in cluster 1; for $n_{\text{clusters}} = 3$, it is 0.5611 with the respective number of persons in each cluster $[71, 36, 39]$; for $n_{\text{clusters}} = 4$, it is 0.5778, with $[52, 36, 36, 22]$; for $n_{\text{clusters}} = 5$, it is 0.5699 with $[42, 36, 34, 22, 12]$, and for $n_{\text{clusters}} = 6$, it is 0.5803 with $[22, 36, 34, 22, 12, 20]$.

building_name	stairs	count	mean	std	min	25%	50%	75%	max
Atrium	back	294.0	0.147462	0.178930	0.0	0.000000	0.064601	0.282223	0.635466
	front	294.0	0.137436	0.220468	0.0	0.000000	0.000000	0.242011	0.799162
Base	back	286.0	0.114866	0.170921	0.0	0.000000	0.000000	0.219620	0.630973
	front	286.0	0.181916	0.243130	0.0	0.000000	0.037269	0.316168	0.815038
Glass	back	310.0	0.039430	0.099490	0.0	0.000000	0.000000	0.000000	0.482952
	front	310.0	0.383792	0.316778	0.0	0.062676	0.358824	0.738898	0.876921

Table S10. Staircase Usage by Building Condition

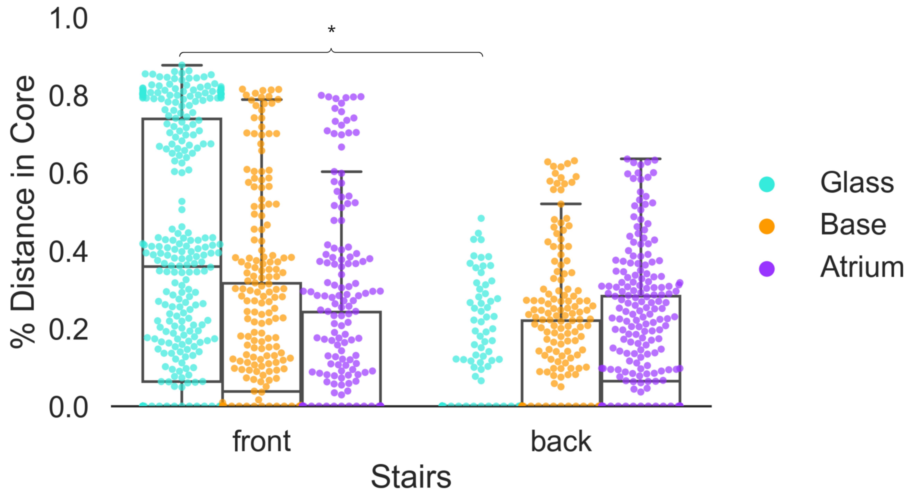

Figure S5. *Utilization of Front and Back Staircases Across Building Conditions*. In the *Glass* group, participants significantly favored the front staircase. While the *Atria* group exhibited a preference for the front staircase, the difference was not statistically significant. In the*Base* condition, the back staircase was used slightly more, but not significantly more than the front staircase. The lack of significance in the *Atria* group (p-value of 0.54) suggests a small effect size (Cohen's d of 0.049).

References

1. Gath-Morad, M., Melgar, L. E. A., Conroy-Dalton, R. & Hölscher, C. Beyond the shortest-path: Towards cognitive occupancy modeling in bim. *Autom. Constr.* **135**, 104131 (2022).
2. Tukey, J. W. Comparing individual means in the analysis of variance. *Biometrics* 99–114 (1949).
3. Heiss, A. Marginalia: A guide to figuring out what the heck marginal effects, marginal slopes, average marginal effects, marginal effects at the mean, and all these other marginal things are (2022). https://www.andrewheiss.com/blog/2022/05/20/marginalia/, Last accessed on 2022-08-31.
4. Munafò, M. R. & Smith, G. D. Robust research needs many lines of evidence (2018).
5. Arel-Bundock, V. *marginaleffects: Marginal Effects, Marginal Means, Predictions, and Contrasts* (2022). R package version 0.7.0.
6. Lüdecke, D. ggeffects: Tidy data frames of marginal effects from regression models. *J. Open Source Softw.* **3**, 772, DOI: 10.21105/joss.00772 (2018).
7. Hanmer, M. J. & Ozan Kalkan, K. Behind the curve: Clarifying the best approach to calculating predicted probabilities and marginal effects from limited dependent variable models. *Am. J. Polit. Sci.* **57**, 263–277 (2013).
8. Duong, T., Goud, B. & Schauer, K. Closed-form density-based framework for automatic detection of cellular morphology changes. *Proc. Natl. Acad. Sci.* **109**, 8382–8387 (2012).
9. Simonoff, J. S. *Smoothing methods in statistics* (Springer Science & Business Media, 2012).
10. Schauer, K. *et al.* Probabilistic density maps to study global endomembrane organization. *Nat. methods* **7**, 560–566 (2010).

11. Anderson, N. H., Hall, P. & Titterington, D. M. Two-sample test statistics for measuring discrepancies between two multivariate probability density functions using kernel-based density estimates. *J. Multivar. Analysis* **50**, 41–54 (1994).
